# What Are the Limits to the Growth of Boreal Fires?

**DOI:** 10.1111/gcb.70130

**Published:** 2025-03-18

**Authors:** Thomas A. J. Janssen, Sander Veraverbeke

**Affiliations:** ^1^ Plant Ecology and Nature Conservation Group Wageningen University Wageningen the Netherlands; ^2^ Faculty of Science Vrije Universiteit Amsterdam Amsterdam the Netherlands; ^3^ School of Environmental Sciences University of East Anglia Norwich UK

**Keywords:** boreal forest, East Siberia, fire, fire growth limitations, fire perimeters, fire weather

## Abstract

Boreal forest regions, including East Siberia, have experienced elevated fire activity in recent years, leading to record‐breaking greenhouse gas emissions and severe air pollution. However, our understanding of the factors that eventually halt fire spread and thus limit fire growth remains incomplete, hindering our ability to model their dynamics and predict their impacts. We investigated the locations and timing of 2.2 million fire stops—defined as 300 m unburned pixels along fire perimeters—across the vast East Siberian taiga. Fire stops were retrieved from remote sensing data covering over 27,000 individual fires that collectively burned 80 Mha between 2012 and 2022. Several geospatial datasets, including hourly fire weather data and landscape variables, were used to identify the factors contributing to individual fire stops. Our analysis attributed 87% of all fire stops to a statistically significant (*p* < 0.01) change in one or more of these drivers, with fire‐weather drivers limiting fire growth over time and landscape drivers constraining it across space. We found clear regional and temporal variations in the importance of these drivers. For instance, landscape drivers—such as less flammable land cover and the presence of roads—were key constraints on fire growth in southeastern Siberia, where the landscape is more populated and fragmented. In contrast, fire weather was the primary constraint on fire growth in the remote northern taiga. Additionally, in central Yakutia, a major fire hotspot in recent years, fuel limitations from previous fires increasingly restricted fire spread. The methodology we present is adaptable to other biomes and can be applied globally, providing a framework for future attribution studies on global fire growth limitations. In northeast Siberia, we found that with increasing droughts and heatwaves, remote northern fires could potentially grow even larger in the future, with major implications for the global carbon cycle and climate.

## Graphical abstract

Fires in East Siberia have been increasing, releasing record greenhouse gas emissions. To understand what stops these fires from spreading, we analyzed fire perimeters from satellite data covering 27,000 fires between 2012 and 2022. We found that fire weather conditions and landscape features, like roads and previous burns, play key roles in limiting fire growth. While human‐altered landscapes in southeastern Siberia help contain fires, remote northern areas are mainly controlled by fire weather. As droughts and heat waves intensify, northern fires could grow even larger, impacting the global climate.

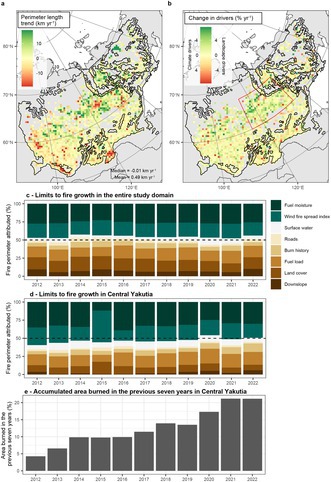

## 1 Introduction

Boreal forests store about one third of all carbon that is sequestered in the world's forest ecosystems, of which more than 60% is stored in soils (Pan et al. 2011, 2024; Tagesson et al. 2020). However, forest plot measurements suggest that the strength of the carbon sink of the world's boreal forests has declined by 36% from the 1990s to the 2010s, mainly resulting from increased fire activity, insect outbreaks, and logging (Pan et al. 2024). Furthermore, greenhouse gas (GHG) emissions, mainly CO_2_ and methane (CH_4_), from permafrost thaw have potentially tipped the scale and turned the Earth's boreal forests into a net carbon source to the atmosphere in recent years (Fan et al. 2023; Treat et al. 2024). Because of their remote location, fires in boreal forests are often ignited by lightning (Kirillina et al. 2020; Scholten et al. 2022; Xu et al. 2022), and while boreal forests are still one of the last places on Earth with extensive intact forest cover, these remote forests are susceptible to lightning fires (Janssen et al. 2023; Potapov et al. 2017). Understanding how the boreal forest carbon balance will respond to climate change, including changing fire dynamics, is crucially important as it might either partly offset anthropogenic GHG emissions and thereby help mitigate climate change or contribute to GHG emissions, thereby further accelerating climate change.

Both fire frequency and intensity have been increasing in boreal forests in the last decades, caused by the warming and drying of boreal forest regions (Gillett et al. 2004; Kasischke and Turetsky 2006; Li et al. 2024). Notably, the years 2019, 2020, and 2021 were the three largest fire years in the Moderate resolution Imaging Spectroradiometer (MODIS) satellite record (since 2001) in eastern Siberia (Scholten et al. 2022). Because of their extensive burned area, these fires contributed to record‐breaking CO_2_ (Zheng et al. 2023) and aerosol emissions (Romanov et al. 2022). Furthermore, these recent extreme fire years are superimposed on a long‐term increasing trend in fire activity in Siberia since the Little Ice Age (after 1850 CE) observed in dendrochronological and sediment records (Kharuk et al. 2016; Novenko et al. 2022). Recent studies overwhelmingly show that future changes in the top‐down drivers of fire activity—a warming and drying climate, earlier snowmelt and associated fuel drying, and more frequent lightning strikes will—compound to more potential boreal fire activity in the future (Hessilt et al. 2022, 2024; Jain et al. 2022; Janssen et al. 2023; Scholten et al. 2022). However, bottom‐up landscape drivers of fire activity might limit future fire growth and may even counteract some effects of the top‐down drivers (Foster et al. 2022; Héon et al. 2014; Parks et al. 2016). Simply put, if there is no fuel left to burn, previous fires will eventually limit the growth of subsequent fires (Parks et al. 2016). Global fire models currently struggle to accurately reproduce fire spread and fire size, which can be largely attributed to a lack of representation of these bottom‐up landscape drivers limiting fire growth (Cardoso et al. 2022; Hantson et al. 2020). While much recent work has focused on the drivers promoting fire growth in boreal forests, either from the perspective of fire weather, fuels, or ignitions, relatively little is known about the mechanisms that constrain it.

The most important top‐down meteorological factors influencing fire spread are air humidity, wind speed, and wind direction. One specific measure of air humidity, the vapor pressure deficit (VPD), which is the difference between the actual and saturated vapor pressure, is strongly linked to fuel drying (Nolan et al. 2016; Viney and Catchpole 1991) and is widely considered a key meteorological indicator of fire ignition efficiency and fire spread potential (Balch et al. 2022; Ray et al. 2005; Sedano and Randerson 2014). In addition to VPD, wind speed and direction have long been recognized as a key drivers of fire spread rates (Byram 1958; Rothermel 1972). Fire spread can accelerate rapidly with increasing wind speeds in the downwind direction as a result of enhanced oxygen supply, the carrying of embers downwind creating new ignitions (i.e., spotting) and, most importantly, the transfer of heat in the downwind direction thereby pre‐heating and drying out fuels (Beer 1991). Inversely, fire spread is limited in the upwind direction because of the same mechanisms. Depending mainly on fuel conditions and terrain slope, fires either do not spread in the upwind direction or at a very slow rate (Byram 1958; Cheney and Sullivan 2008; Weise and Biging 1996). Therefore, both a sudden decline in wind speed and a change in wind direction might significantly limit fire growth in the direction that the fire was previously heading.

Besides top‐down controls, bottom‐up landscape controls related to fuels and topography also influence the spread of fires. Landscape fragmentation, either natural or anthropogenic, limits fuel connectivity and constrains burned area and fire size globally (Haas et al. 2022). Fragmentation through natural fire barriers includes different types of wetlands and water bodies where fuel is either too wet to burn or completely absent (Cyr et al. 2005; Erni et al. 2017; Gromtsev 2002; Madoui et al. 2010; Nielsen et al. 2016). Anthropogenic fire barriers that contribute to fragmentation are mostly represented by roads and fuel‐limited anthropogenic land cover such as croplands (Andela and van der Werf 2014; Haas et al. 2022; Kuklina et al. 2023). Importantly, previously burned areas also limit fuel connectivity and can be considered either natural or anthropogenic depending on the ignition source (Erni et al. 2018; Héon et al. 2014; Parks et al. 2016). Topography is also known as an important driver of fire growth (Eftekharian et al. 2019; Liu 2023; Pyne et al. 1996). Fires generally spread upslope due to the preheating of fuels and wind enhancement effects on combustion (Eftekharian et al. 2019; Liu 2023). Conversely, downslope fire spread is generally reduced due to the lack of fuel preheating and canceling out wind enhancement effects (Eftekharian et al. 2019; Pyne et al. 1996; Sullivan et al. 2014; Weise and Biging 1996). It is presently unknown which and to what extent these different weather and landscape constraints limit fire growth in boreal forests and how these will change in the future, especially in the remote east Siberian taiga.

We examined fire perimeter outer boundaries (hereafter, ‘fire stops’) to investigate how weather and landscape factors impede fire spread. We used satellite‐derived burned area and active fire data from East Siberia spanning 2012 to 2022 to determine fire stops at a high spatial resolution of 300 m and a temporal resolution of 1 day. Multiple geospatial datasets based on remote sensing and climate reanalysis were used to match the location and timing of fire stops to potential top‐down weather influences or bottom‐up landscape constraints. Overall, we aim to better understand the complex interactions between climate and landscape drivers in both promoting and limiting fire growth in boreal forest ecosystems. Our findings can inform fire management strategies by identifying key weather and landscape conditions that constrain fire spread, thereby improving predictive models and risk assessments for boreal regions.

## 2 Methods

### 2.1 Study Domain

**FIGURE 1:**
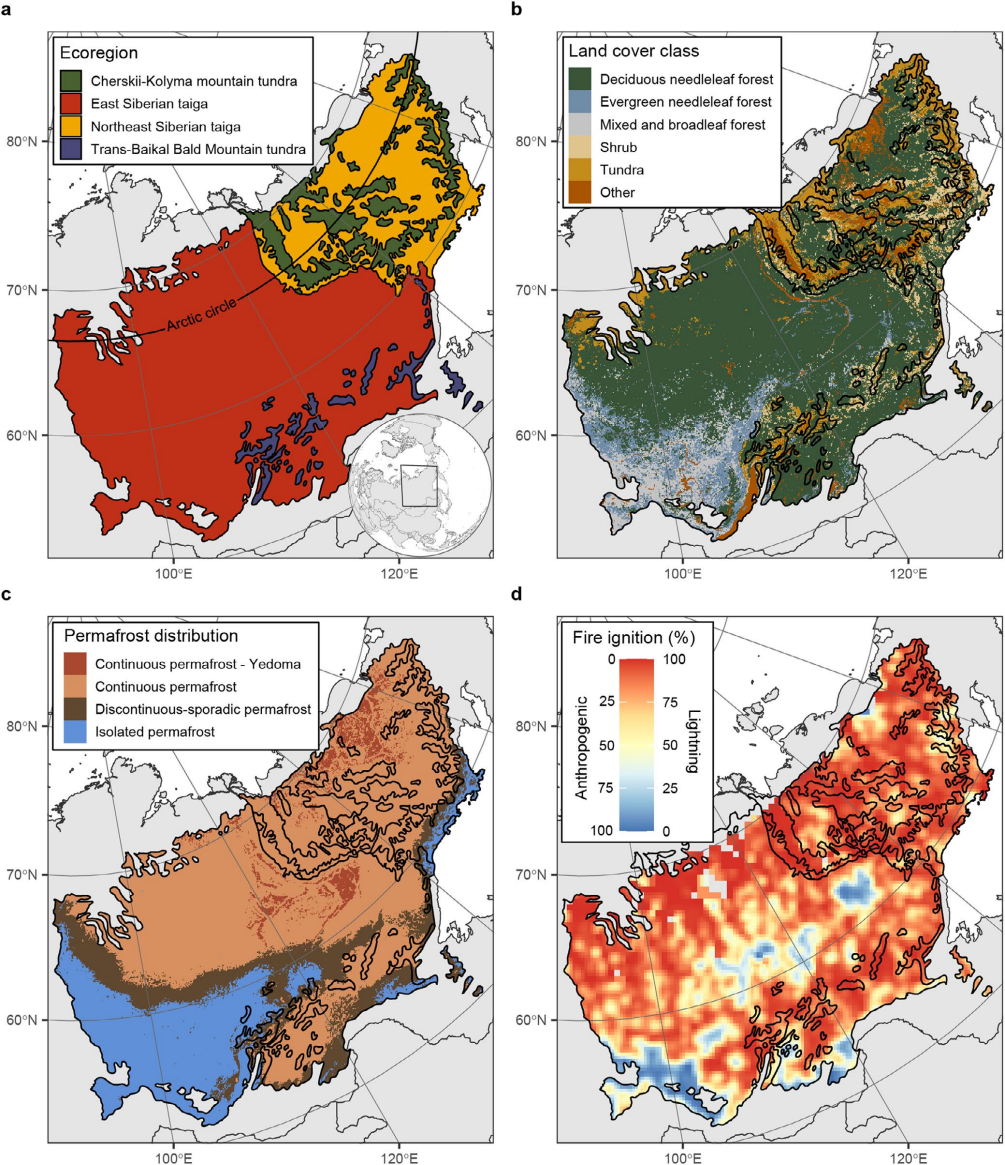
Overview of the study domain with (a) ecoregions, (b) land cover, (c) permafrost landscapes, and (d) fire ignition sources. The inset in the lower‐right corner of panel (a) shows the location and extent of the study domain. The grey lines denote country boundaries and the black lines ecoregion boundaries. Ecoregions are derived from Olson et al. (2001), land cover is adapted from Bartalev et al. (2003, 2010), permafrost distribution is derived from Obu et al. (2019), Yedoma presence is from Strauss et al. (2022) and ignition sources are from Janssen et al. (2023). The landcover and permafrost distribution were both aggregated by majority class to a 5 × 5 km spatial resolution for easier visual interpretation, the ignition source data was maintained at its original 0.5° spatial resolution. See Supporting Information Methods for a detailed description of the datasets. Map lines delineate study areas and do not necessarily depict accepted national boundaries.

Our analysis considered the fire‐prone eastern Siberian boreal forest, which includes the Siberian taiga and mountain tundra ecoregions (Olson et al. 2001). We selected the East‐ and Northeast Siberian taiga ecoregions as well as the adjacent Cherskii‐Kolyma Mountain tundra and the Trans‐Baikal Bald Mountain tundra ecoregions (Figure 1a). These four ecoregions combined cover an area of 5.9 million km^2^. The northern taiga is largely covered by deciduous needle leaf forest (67%), dominated from west to east by Siberian larch ( *Larix sibirica* ), Dahurian larch ( *Larix gmelinii* ), and Cajander larch (*Larix cajanderi*) (Abaimov 2010). In the south, the vegetation consists mainly of evergreen needle leaf and mixed needle leaf‐broad leaf forest (15%, Figure 1b). Dominant evergreen needle leaf and broad leaf tree species include Siberian pine ( *Pinus sibirica* ), Scots pine ( *Pinus sylvestris* ), Siberian spruce (*Picea obovate*), Siberian fir (*Abies sibirica*), and silver birch ( *Betula pendula* ). The mountain tundra ecoregions consist of gradients and mosaics of different vegetation types, mainly deciduous needle leaf forest (38%), tundra (35%), and shrub (12%, Figure 1b).

Most of the study domain is underlain by continuous permafrost soils (64%) of which 7% consists of deep organic‐rich Pleistocene soils with a high ice volume content (more than 50%), known as Yedoma (Figure 1c). In about 16% of the domain, the permafrost cover is discontinuous or sporadic (between 10% and 90% cover), while in the remaining 20% of the study domain, mainly in the southwest, permafrost does not occur or only occurs as isolated patches (less than 10% cover). These remote taiga and tundra ecoregions are dominated by lightning‐ignited fires, which are responsible for 82% of the burned area (Figure 1d). Human‐ignited fires are dominant only in specific regions, primarily in the center of the study area and along the southern edges where human population densities are higher (Figure S1a,b). Most fires in these regions are large wildfires, burning between 1% and 10% of the land area annually (Figure S1c).

### 2.2 Data

To delineate fire perimeters, we harmonized three different burned area and active fire datasets: (1) the European Space Agency (ESA) Fire Climate Change Initiative FireCCI51 250 m burned area product, (2) the Copernicus Climate Change Service (C3S) 300 m burned area product, and (3) the 375 m resolution Visible Infrared Imaging Radiometer Suite (VIIRS) active fire dataset (see Supporting Information Methods for details). From the harmonized burned area data, we retrieved 2.5 million fire stops, representing a total perimeter length of 749,000 km. The fire perimeter density was 45.4 m km^−2^ year^−1^ across the study domain, with higher densities of up to 350 m km^−2^ year^−1^ in the human‐dominated southern parts of the study area (Figure S1d). Perimeters from more than 27,000 individual fires were included in the analysis that burned on average 7.2 Mha year^−1^. About 90% of these fires were recorded in the months of April to September (Figure S1e). However, there were large spatial differences in fire seasonality. In the southern part of the study domain, spring fires prevail, while summer fires dominate the remaining area. The mean fire size in our study domain was 11.3 km^2^, while the median fire size was relatively small (0.54 km^2^). Yet, some remote areas are characterized by very large median fire sizes (larger than 100 km^2^, Figure S1f).

We used several geospatial datasets, including hourly fire weather and landscape data, to derive the causes of fire stops (Table 1). The burned area data handling and the handling of the geospatial variables are described in detail in the Supporting Information Methods.

**TABLE 1:** Potential drivers limiting fire growth. Meteorological data was derived from the 5th generation European Centre for Medium‐Range Weather Forecasts (ECMWF) reanalysis land product (ERA5‐Land, Muñoz Sabater 2019). Above ground biomass and the burned area products were obtained from the European Space Agency Climate Change Initiative (ESA CCI, FireCCI) and the Copernicus Climate Change Service (C3S). The active fire data was retrieved from the Visible Infrared Imaging Radiometer Suite (VIIRS) VNP14IMGM data product. Elevation data was obtained from the Advanced Land Observing Satellite (ALOS) Global Digital Elevation Model (DEM). Data on road networks were derived from the Global Roads Inventory Project (GRIP), the Global Roads Open Access Data Set (gROADS), and Open Street Map (OSM).

Type	Potential driver	Temporal resolution	Spatial resolution	Data type	Test	Source
Fire weather drivers	Wind fire spread index	Hourly	0.1°	Numerical	Breakpoint + *t*‐test	ERA5‐Land (Muñoz Sabater 2019)
	Vapor pressure deficit	Hourly	0.1°	Numerical	Breakpoint + *t*‐test	ERA5‐Land (Muñoz Sabater 2019)
	Surface soil moisture content (0–7 cm)	Hourly	0.1°	Numerical	Breakpoint + *t*‐test	ERA5‐Land (Muñoz Sabater 2019)
Landscape drivers	Tree cover	Yearly	30 m	Numerical	*t*‐test	Global forest change (Hansen et al. 2013)
	Above ground biomass	Yearly	100 m	Numerical	*t*‐test	ESA CCI Biomass project (Santoro et al. 2021)
	Land cover class	—	345 m	Categorical	*χ* ^2^ test	Land cover map of Russia
	Burn history	Yearly	300 m	Numerical	*t*‐test	Harmonized burned area data (FireCCI + C3S + VIIRS)
	Terrain slope	—	30 m	Numerical	*t*‐test + 5° slope threshold	ALOS Global DEM (Takaku et al. 2020)
	Road presence	—	30 m	Categorical	*χ* ^2^ test	GRIP, gRROADS and OSM
	Surface water presence	Yearly	30 m	Categorical	*χ* ^2^ test	Global surface water (Pekel et al. 2016)

### 2.3 Analyses

#### 2.3.1 Obtaining Fire Perimeter Locations

**FIGURE 2:**
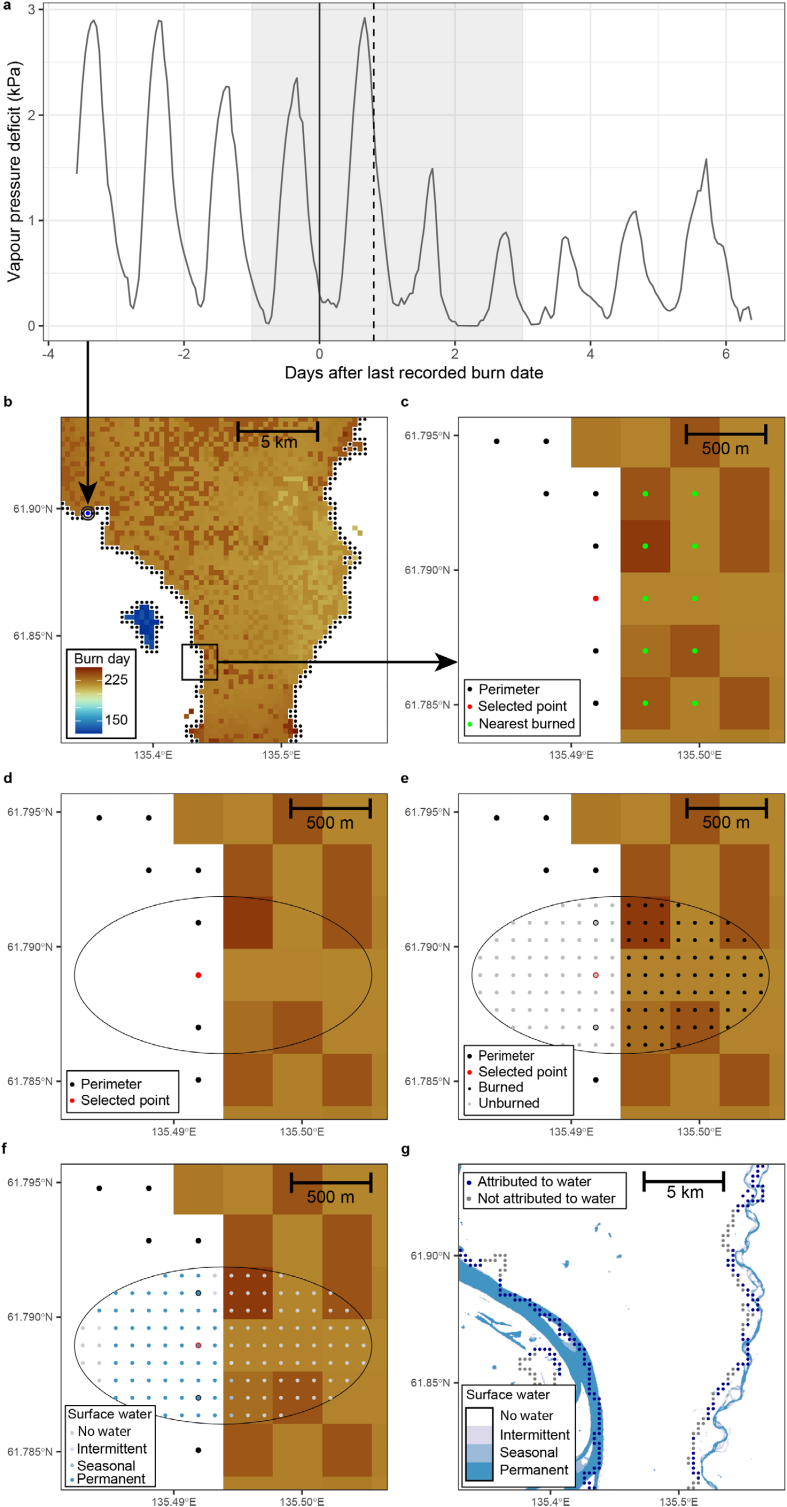
Attributing top‐down fire weather and bottom‐up landscape drivers to fire stops in a subset of the study domain. (a) Timeseries of vapor pressure deficit (VPD) surrounding the last recorded burn date at a single fire stop showing a structural break (dashed line) and a decline in VPD at the last recorded burn date. The grey shaded area depicts the 4‐day period surrounding the last recorded burn date in which a structural break in a fire weather variable might result in that variable being attributed to the fire stop. (b) The harmonized burned area data of the year 2020 and the perimeter locations or fire stops. (c) Establishing the direction perpendicular to the fire perimeter by identifying the nearest burned pixels (d) using the direction perpendicular to the fire perimeter to determine the orientation of the ellipse (e) creating a 100 by 100 m regular grid of burned and unburned spatial point locations inside the elliptical polygon to extract landscape variables (f) extracting the global surface water values to the grid. (g) The global surface water data and the perimeter locations with surface water attribution.

The focus of our analyses is on the fire perimeter outer boundary. To be able to analyze the factors resulting in fire cessation on a continuous perimeter, we segmented the perimeters into multiple equally spaced fire perimeter locations or fire stops. These fire stops represent a single spatial point along the fire perimeter to which we can assign a potential cause of the fire stopping there at a specific time (Figure 2). Before we obtained these locations, we omitted fires smaller than six contiguous pixels (0.54 km^2^, which was also the median fire size) from the dataset because our focus was on large wildfires. Subsequently, for each year in the harmonized burned area dataset (2012–2022), we buffered the burned area pixels (300 m) by a single pixel following first‐order Queen's adjacency so that a at least 300‐m‐wide buffer surrounded all observed fire pixels. These pixels were then converted to spatial point locations with a latitude and longitude coordinate, the fire perimeter locations (Figure 2). We then extracted the burn date of the nearest burned pixel to obtain the final burn date before fire cessation at the perimeter. This procedure yielded on average per year around 227,000 fire stops (68,000 km) from 2481 fires, with up to 346,000 fire stops in the record year 2020.

#### 2.3.2 Attributing Limitations to Fire Spread

The perimeter locations and their associated last recorded burn date indicate the location and timing of fire stops, yet not their potential causes. To attribute the causes of the fire stops, we carried out two types of analyses. In these two analyses, every separate potential driver (Table 1) could be flagged as a cause, so that multiple causes for a single perimeter point were possible.

The first analysis was carried out by using the high temporal resolution of the ERA5 Land‐derived meteorological variables (Muñoz Sabater 2019) in a time series analysis. At each perimeter point, we extracted the hourly VPD, wind fire spread index (WFSI), and surface soil moisture data for the 10 days surrounding the last recorded burn date (Figure 2a). The WFSI is a newly developed index for this study based on hourly wind direction and wind speed that indicates whether the wind is aligned with the fire front (positive values) or opposing the fire front (negative values) and scaled by the wind speed (see Supporting Information Methods for details). For all three variables, we first performed a Student's *t*‐test using the R package *stats* to establish whether there was a significant (*p* < 0.01) difference between the 5 days before and including the last recorded burn date and the 5 days after the last recorded burn date. If a significant decline in VPD and WFSI or a significant increase in soil moisture were observed that could indicate a cause for the fire stop, we proceeded to the next step. In the next step, to detect the presence of a significant sudden change or breakpoint in the time series, we used the method developed by Bai and Perron (2003) and implemented in the R package *strucchange* to derive single or multiple break points in the time series. If a breakpoint was detected and if the breakpoint (including its confidence interval) was timed 1 day before to 2 days after the last recorded burn date, we flagged the perimeter point as potentially caused by a change in the specific weather variable (Figure 2a).

The second analysis leveraged the high spatial resolution of the landscape variables to establish whether there was a significant change of a variable in space along a gradient from inside to outside of the fire perimeter. For this analysis, at each fire perimeter point, an ellipse was created and oriented with its major axis perpendicular to the fire perimeter (Figure 2b–d). The major axis length was set to three times and the minor axis length to one and a half times the spacing between the fire perimeter points, which is the same as the spatial resolution of the harmonized burned area data (300 m). The center of the ellipse was set halfway in between the perimeter point location (the centroid of the first non‐burned pixel) and the nearest burned pixels so that approximately half of the area of the ellipse overlapped with burned pixels and the other half overlapped with unburned pixels (Figure 2e).

**FIGURE 3:**
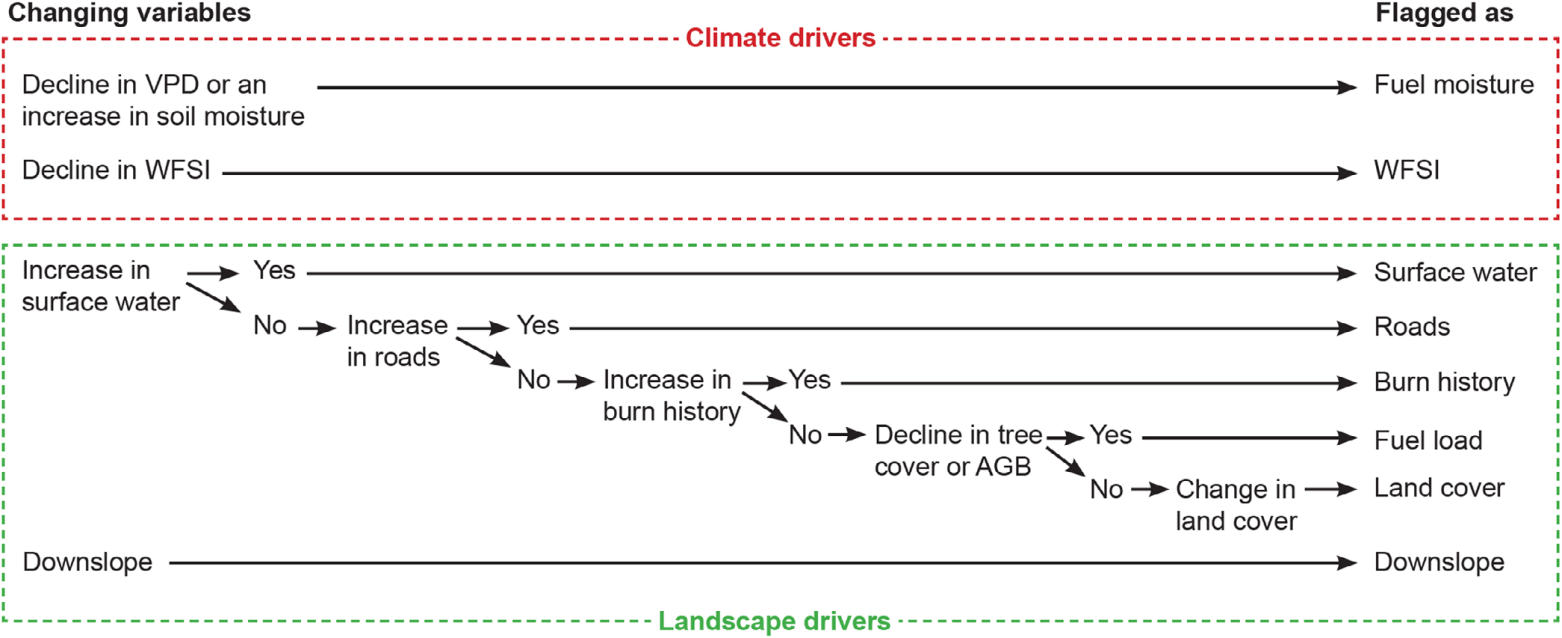
Attributing changing climate and landscape variables as potential drivers of fire cessation using a decision tree model. The effect of a change in the climate drivers related to fuel moisture, that is, a decline in vapor pressure deficit (VPD), an increase in soil moisture or both, and the wind fire spread index (WFSI) were tested using a time series analysis. The effects of changes in the landscape drivers were tested separately as a change in these variables over space from inside to outside the fire perimeter (see Figure 2).

Subsequently, the data for all landscape variables were extracted to the ellipse at a 100 m resolution spacing, a spatial resolution in between the highest resolution landscape datasets (30 m) and the lowest spatial resolution land cover map (345 m, Table 1). In that way, we obtained two groups with observations of landscape variables representing two halves of the elliptical polygon, a group of burned locations inside the perimeter and a group of unburned locations located outside the perimeter (Figure 2f). We performed two types of tests to establish a statistically significant difference in the landscape variables between these groups (see Supporting Information Methods for more details on the variables). For continuous variables such as above‐ground biomass (AGB), percentage tree cover, and burn history, we performed a *t*‐test. For the categorical variables such as land cover type and the presence of roads and water, we performed a *χ* ^2^ test. In case of a statistically significant difference (*p* < 0.01) between the groups for road and water presence, we also confirmed that the road and water presence were in fact higher outside the perimeter by comparing the means of the groups. Similarly, we confirmed that if a significant difference in land cover was detected, the relatively highest increase in the area cover of a land cover class outside the perimeter was different than the most common land cover class inside the fire perimeter. For downslope, we tested whether there was a significant decline in elevation from inside to outside the perimeter and only flagged perimeter locations as being caused by downslope if the slope was lower or equal to −5°, which is a threshold value found in experimental studies below which downslope substantially limits fire spread (Sullivan et al. 2014). Finally, we combined the variables VPD and soil moisture into the variable *fuel moisture*, which is defined as a significant decline in VPD and/or an increase in soil moisture surrounding the last burn date, signaling a fuel rewetting event (Figure 3). We also combined the variables tree cover and AGB into the variable *fuel load*, as a significant decline in one or both of these proxies from inside to outside the fire perimeter could point to a limitation in above‐ground fuel load (Figure 3).

In our analysis, multiple potential causes can be attributed to a single fire stop. Fire quenching often results from the combined influence of multiple drivers. For example, barriers such as roads and rivers may not effectively stop fire spread unless weather conditions change simultaneously, such as a drop in wind speed or an increase in humidity, at the moment the fire encounters the barrier (Holsinger et al. 2016; Moreno et al. 2014). However, our list of potential drivers included some drivers that supersede the effect of other drivers. For example, if a fire is extinguished at the banks of a large river, in our analysis, this location will be flagged as caused by a change in surface water, a change in fuel load, and a change in land cover. To prevent this double counting, we used a simple hierarchical decision tree to separate these interdependent drivers (Figure 3). The two fire‐weather drivers, an increase in fuel moisture, and a decline in the WFSI could always be flagged separately as they can be considered independent from each other and independent from the landscape drivers. Also, the presence of a pronounced downslope in the landscape is independently flagged as a potential cause (Figure 3). The remaining landscape drivers were all related to the availability of fuels, and therefore, these were hierarchically ordered from relatively hard fire barriers such as water bodies to more subtle landscape changes. The presence of surface water (1) and roads (2) were considered hard barriers, offering no available fuel. Hereafter, the burn history (3) was chosen in the decision tree as a previous fire would have substantially impacted the availability of fuels (4) as well as the land cover (5). The availability of fuels (4) was chosen over a change in land cover (5) as a decline in fuel load is directional, while for a change in land cover, we only test for a significant difference in land cover and not a change to a less flammable land cover per se. In this way, at each fire stop, a maximum total of four independent drivers could be flagged as a potential cause (Figure 3). Because of this, for most statistics and figures, we used the percentage of perimeter points where an independent driver was flagged, which therefore could add up to a total of more than 100%. For the figures showing relative contributions (e.g., stack bars) where the total must add up to 100%, we divided the contribution of every driver by the total number of flagged drivers at each fire stop.

To be able to visualize our results as maps over the entire study domain (5.9 million km^2^) and in order to obtain area‐averaged statistics, we spatially aggregated the results of the pixel‐based fire stop analysis. For all maps and spatial analyses, we used the Lambert azimuthal equal‐area projection for Russia (EPSG:3576). For displaying the spatial patterns over the entire study period, we aggregated to a 20 km grid (400 km^2^ cells). For the time series analysis, to be able to have enough perimeter observations per year, we aggregated the data to a 40 km grid (1600 km^2^ cells). All trend analyses were performed using the ordinary least squares (OLS) regression function from the R package *stats*.

## 3 Results

### 3.1 A Proof of Concept

**FIGURE 4:**
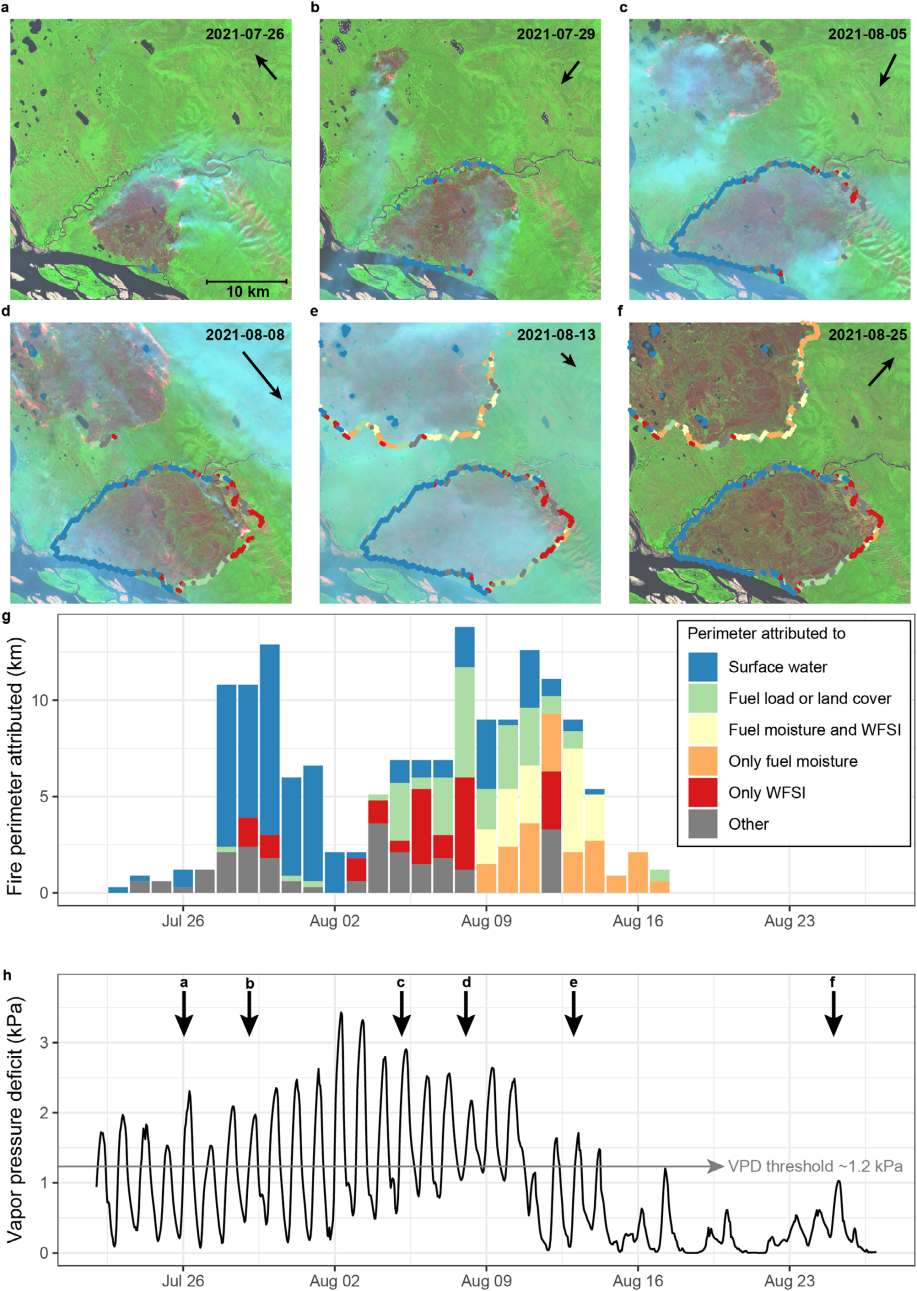
A demonstration of the methodology on two averaged size fires that burned in 2021 along the Lena River, 350 km northwest of Yakutsk, Russia. Panels (a–f) show the progression of burning using a Sentinel‐2 false colour composite to visualize the active fires, smoke plumes, and burned area. The acquisition dates are at the top of the panel, with an arrow representing the wind direction and speed derived from ERA5‐Land hourly reanalysis data. In panels (a–f), the fire stops on and before the date of image acquisition are plotted with the colours representing which subset of potential drivers the point was attributed. (g) The daily sum of fire stops attributed to a subset of potential drivers and (h) the time series of average vapor pressure deficit (VPD) at 2 m in the area derived from ERA5‐Land reanalysis data, with the image acquisition dates indicated with the downward pointing arrows and associated panel letters. The grey horizontal arrow indicates that the 1.2 kPa VPD threshold below which fire activity is found to be extremely rare in these boreal ecosystems, derived from Balch et al. (2022) and Clarke et al. (2022).

To provide a proof of concept of our method, a small subset of the study domain was selected for a detailed analysis on how the algorithm performed on two average‐sized fires that burned for about 1 month in 2021 (Figure 4). Because of limited cloud cover in this period, we were able to use six high‐quality Sentinel‐2 images to track and visualize the fire spread and the development of the fire perimeter. At the end of July 2021, the most southern fire is spreading while confined by the Lena River in the south and a smaller tributary in the northwest (Figure 4a–c). On the 8th of August, the outer perimeter of the southernmost fire is almost complete, while the northern fire is still spreading (Figure 4d). Finally, on the 25th of August, both fires have been completely quenched with no new burned area (i.e., both fires stopped spreading, Figure 4f). While the distance between the outer perimeters of both fires is only 5 km, the attributed drivers impeding fire spread were very different. In the early southern fire, most of the perimeter was attributed to bottom‐up landscape drivers, mostly the presence of surface water, and, to some degree, to a decline in fuel load and differences in land cover (Figure 4f). This is in contrast to the later northern fire that was largely quenched by a top‐down increase in fuel moisture (Figure 4f). This transition can also be observed in the time series of fire perimeter attribution (Figure 4g), which starts off as heavily dominated by the presence of surface water before the 2nd of August, followed by a mix of landscape drivers and a decline in the WFSI and after the 8th of August by fuel moisture as VPD declined. In general, despite the difference in spatial resolution between the harmonized burned area data (300 m) from which the perimeters were derived and the Sentinel‐2 images (20 m), the algorithm performs well in ascribing large parts of the fire perimeter to the presence of water bodies, both in the form of rivers and streams in the southern fire as well as solitary lakes in the northern fire.

### 3.2 Importance of Different Drivers Impeding Fire Growth

**FIGURE 5:**
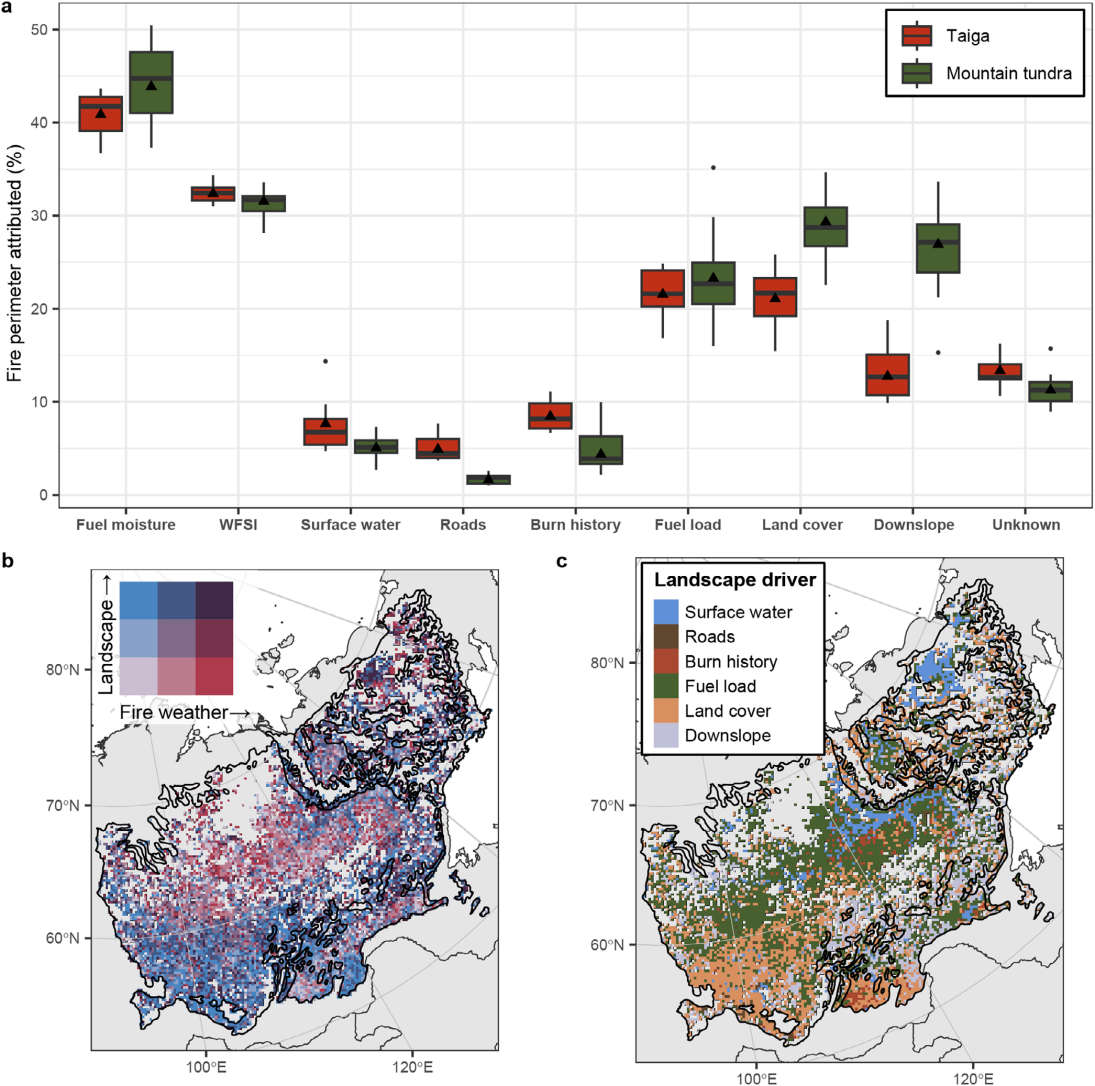
The contribution of different drivers to limiting fire growth in eastern Siberia. (a) The contribution of the examined potential drivers of fire cessation, (b) the relative importance of the fire weather drivers and the landscape drivers in limiting fire growth, and (c) the most important landscape driver limiting fire growth. The box plots show the annual contribution for the 11 years (2012–2022) of the analysis with the interannual mean shown as black triangles. The maps (b, c) were created by spatially aggregating the pixel‐based fire stop results to a 20 km resolution grid using a Lambert azimuthal equal‐area projection. Map lines delineate study areas and do not necessarily depict accepted national boundaries.

The complete analysis yielded 2.5 million fire stops (each representing an individual 300 m pixel), corresponding to 749,000 km of fire perimeter covering eastern Siberia over a period of 11 years (2012–2022). For 87% of these fire stops, we found a possible cause impeding fire spread using a conservative significance level (*p* < 0.01), leaving 13% unexplained (Figure 5a). We found that 28% of all fire stops were caused by one of the landscape drivers, 20% were linked to one of the fire‐weather drivers, and 39% were caused by both fire weather and landscape. The most important limitation to fire growth is fuel moisture (Figure 5a) with approximately 41% (interannual standard deviation = 2.3%) of the total fire perimeter length explained by a sudden increase in fuel moisture at the time of fire cessation. Of these fire stops explained by an increase in fuel moisture, 32% was associated with a decline in VPD, 31% to an increase in soil moisture, and the remaining 37% to a simultaneous change in both. The second most important driver, a decline in the WFSI (Figure 5a), was flagged as a potential cause at 32% (± 1.0%) of the total fire perimeter. More than half of all fire stops (58% ± 2.2%) were attributed to a change in these two fire‐weather drivers, either to only an increase in fuel moisture (26%), only a decline in WFSI (17%), or a simultaneous change in both (15%). When comparing the relative contribution of the fire‐weather drivers and the landscape drivers to limiting fire growth across the study domain, we found that the fire‐weather drivers dominate the northwestern part of the study domain (> 60° N), while landscape limitations are the most important in the southwest (Figure 5b).

Of the six bottom‐up landscape drivers, we found a relatively small contribution of the surface water presence in explaining fire stops, with only 7% (± 2.7%) of the perimeters explained by an increase in surface water (Figure 5a). However, surface water was identified as the most important cause of fire stops in the landscapes dotted by numerous thermokarst lakes associated with ice‐rich Yedoma deposits, especially in the Kolyma Lowland in the northeastern part of our study domain (Figure 5c, Figure S3). In this region, about half (47%) of the fire stops in the exceptionally severe fire season of 2020 were caused by the presence of water bodies (Figure S3). Roads acting as fire barriers generally also contribute little, explaining only 5% (± 1.2%) of the fire stops across the study domain. Fuel reduction because of a previous fire (burn history) was also a relatively minor limitation on fire growth, as it explained only 8% (± 1.5%) of all fire stops (Figure 5a). However, burn history was identified as the most important landscape driver limiting fire growth in the frequently burned southern edges of the study domain (Figure 5c). A decline in above‐ground fuel load and a land cover transition at the perimeter were the most important landscape drivers impeding fire growth, explaining 22% (± 2.6%) and 22% (± 2.8%) of all fire stops, respectively. Fuel load was the most important landscape driver across 38% of the study domain, predominantly in the north (> 60° N) of the East Siberian taiga ecoregion (Figure 5c). In contrast, land cover was the most important landscape driver in 29% of the study domain, mainly in the southwest (< 60° N) and in the mountain tundra ecoregions, which consist of patchier landscapes with evergreen needle leaf, mixed forest, and shrub vegetation (Figure 1b, Figure 5a,c).

We found that almost half (48%) of the fire stops were attributed to a transition in land cover; a transition occurred from deciduous needle leaf forest (*Larix* spp.) inside the fire perimeter to another land cover class (mainly evergreen light needle leaf forest, mixed forest, and deciduous broad leaf forest) outside the perimeter (Figure S4). Furthermore, the land cover classes mixed forest and evergreen dark‐ and light needle leaf forest were all at least twice as likely to occur outside than inside the fire perimeter, suggesting that these land cover classes often act as fire stops (Figure S4a). The inverse applies to the land cover classes deciduous needle leaf forest and humid grasslands, which were twice as likely to occur inside than outside the fire perimeter, suggesting that these land cover classes are conducive to fire spread.

The two continuous landscape variables, burn history and fuel load, also showed clear differences between inside and outside the perimeter (Figure S5). At the perimeter locations that were attributed to burn history, the fuel load reduction due to a previous fire was higher outside the perimeter (16% fuel load reduction) compared to inside (4% fuel reduction) with a mean decline of 12% fuel reduction across these locations (Figures S5a,b). The two proxies for fuel load, tree cover and AGB, showed similar trends when going from inside to outside the perimeter (Figure S5). At the fire stops that were attributed to fuel load due to a decline in tree cover, pre‐fire tree cover inside the perimeter was substantially higher (45%) than outside the perimeter (26%) with a mean decline of 15% (Figure S5c,d). Similarly, the pre‐fire AGB was higher inside the perimeter (48 Mg ha^−1^) compared to outside (23 Mg ha^−1^) with a mean decline of 24 Mg ha^−1^ when going from inside to outside the perimeter (Figure S5e,f).

Finally, another moderately important fire growth limitation was the presence of a pronounced (more than 5°) downslope in the relief, explaining 14% (± 3.1%) of the perimeters (Figure 5a). Not surprisingly, the relative contribution of downslope to explaining the fire perimeter location was twice as large in the mountain tundra ecoregions (26% ± 5.0%), where slopes play a larger role in fire behaviour, compared to the relatively flat taiga ecoregions (13% ± 3.0%). Downslope was the most important landscape driver across 20% of the study domain, primarily in the southeastern and eastern parts of the domain (Figure 5c).

### 3.3 Spatial Variability in the Importance of Drivers Impeding Fire Growth

**FIGURE 6:**
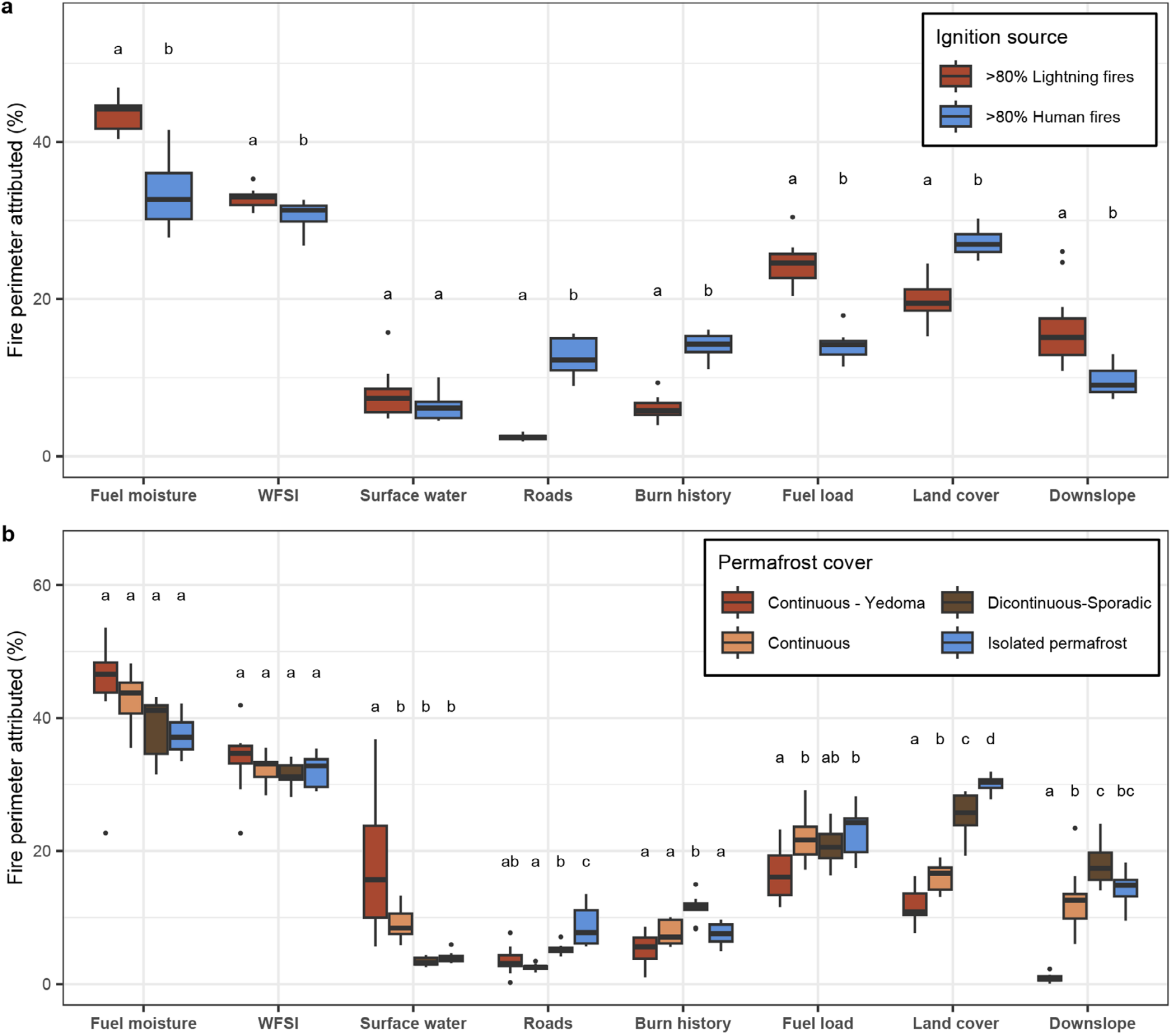
Spatial variability in the drivers of fire cessation in northeastern Siberia grouped by (a) the dominant fire ignition source (human or lightning) and (b) the cover of permafrost soils and Yedoma. The boxplots show the annual contribution for the 11 years (2012–2022) of data. Different letters denote groups that are significantly different (*p* < 0.01) from each other using a Tukey post hoc test.

Clear differences in the contribution of fire growth limitations emerge when looking at different fire regimes and dominant ignition sources (Figure 6a). First, an increase in fuel moisture is a more important constraint on fire growth in areas where natural lightning fires dominate (43% ± 2.1%) compared to areas where human fires dominate (33% ± 4.4%). On the other hand, roads are relatively important fire stops in areas dominated by human ignitions (13% ± 2.3%) and roads rarely constitute a fire stop in lightning‐dominated areas (2% ± 0.3%). Furthermore, because fires occur more often in areas with human ignitions (Figure S1a), the fuel limitations imposed by burn history explained more than double the percentage of fire stops in these areas (14% ± 1.7%) compared to areas dominated by lightning ignitions (6% ± 1.5%). More strikingly, we found that the importance of fuel load and land cover switches places as the most important landscape drivers when comparing lightning and human‐dominated fire regimes. The more fragmented landscapes dominated by human ignitions show a lower contribution of fuel load (15% ± 1.8%) and a higher contribution of land cover change (27% ± 1.7%) in explaining fire stops, compared to the more intact continuous forests where lightning ignitions dominate and where fuel load is the most important landscape driver (24% ± 2.7%) and land cover is less important (20% ± 2.6%). Finally, the downslope limitation was more important in the remote lightning‐dominated fire regimes (16% ± 5.0%) compared to the human‐dominated fire regimes (10% ± 2.0%). For clarity, these differences in the importance of different drivers between human and lightning‐dominated fire regimes do not necessarily imply that drivers become more or less *effective* in stopping fire spread or that human and lightning fires behave differently. For example, roads are probably as effective as fire breaks in lightning‐dominated regimes as they are in human‐dominated fire regimes, but the density of roads is much lower in areas dominated by lightning fires, and therefore, the probability of a fire being stopped by a road is also lower in these areas. We can, however, conclude from our analysis that in areas with different fire regimes and ignition sources, the relative importance of different drivers impeding fire growth differs as well.

As permafrost soils are of major importance for the global carbon cycle and regional hydrology, we also examined the differences in the contribution of drivers impeding fire growth in different permafrost regions. We found that the contribution of surface water in explaining fire stops is substantially higher in areas with Yedoma‐type permafrost soils (18% ± 10.5%) compared to the other regions (5% ± 2.9%). Yedoma landscapes are often characterized by a high density of thermokarst lakes and thaw ponds, providing a high density of potential fire stops. Generally, we found an increasing importance of roads, burn history, and land cover transitions with a decline in permafrost cover (Figure 6b). Because these are all related to the often human‐caused fragmentation of the landscape, it is likely that these trends reflect the strong spatial co‐occurrence of human settlements, human ignitions, and the absence of permafrost soils.

As we obtained and attributed fire stops for individual fires (Figure 4), we also assessed the relative importance of the different drivers as a function of fire size (Figure S6). The relative importance of the different drivers of fire growth remained relatively stable when going from very small fires (smaller than 3 km^2^) to very large fires (larger than 900 km^2^), suggesting that the growth of both small and large fires is often limited by the same drivers. However, we found some subtle differences in the relative importance of the drivers limiting fire growth as a function of fire size. Most notably, the importance of land cover declined gradually with increasing fire size (Figure S6a), explaining 20% of the perimeter of fires in the smallest size class (1–3 km^2^) and only 8% of the perimeter of fires in the largest size class (larger than 902 km^2^). Similarly, although generally of minor importance, roads were attributed to 1.5% of the fire stops in the smallest fire size class and to only 0.5% in the largest size class. The relative decline in the importance of land cover and roads in explaining fire stops with increasing fire size was counteracted by the increasing relative importance of fuel moisture (24%–27%), fuel load (9%–14%), and surface water (4%–9%) when going from the smallest to the largest fires (Figure S5a). We also found that a large majority (75%) of the fire stops analyzed in our study originated from small to medium‐sized fires (0.5–283 km^2^) while more than half of the burned area stems from large (larger than 283 km^2^) fires (Figure S6b).

### 3.4 Seasonal and Daily Dynamics of Drivers Impeding Fire Growth

**FIGURE 7:**
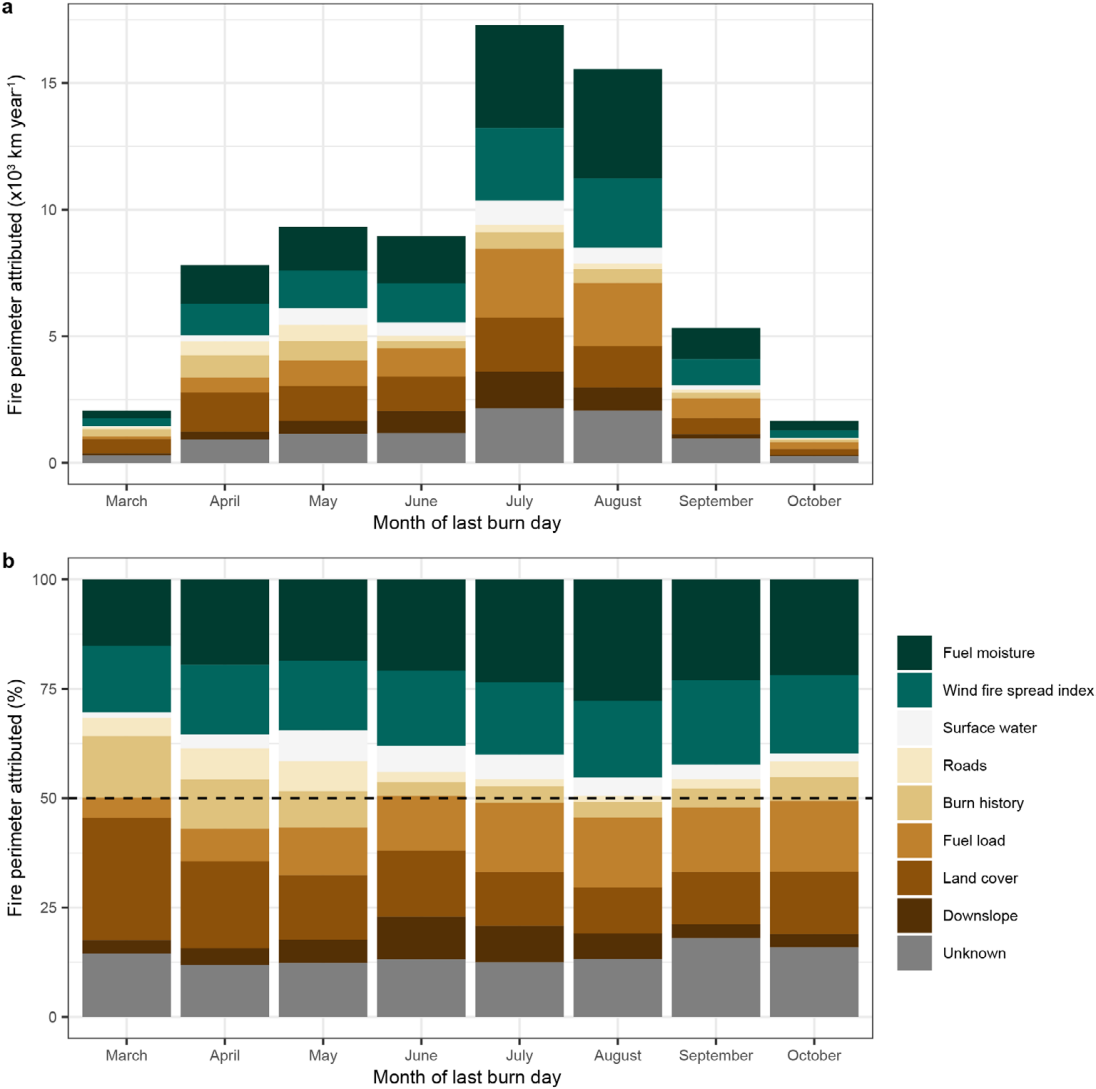
Seasonal changes in the contribution of different potential drivers of fire cessation. (a) The contribution to all fire stops expressed as the represented perimeter length and (b) the relative contribution expressed as the percentage of the total perimeter explained in that month.

We found that the contribution of the different potential drivers impeding fire growth changed over the course of the fire season (March–October, Figure 7). Notably, the relative contribution of the drivers related to fire weather (fuel moisture and WFSI) both increased from the early fire season in March, when together they explain 30% of the fire stops, to the peak of the fire season in August, when they explain nearly half (45%) of the fire stops (Figure 7b). The inverse is true for the combined contribution of roads, burn history, and land cover, which steadily decline from explaining 46% of the fire stops in March to only 15% in August. The contribution of the remaining three drivers—surface water, fuel load, and downslope—all increased over the course of the fire season, from a combined contribution of 9% in March to 26% in August (Figure 7b). These results suggest that the early‐season fires show different fire spread dynamics compared to late‐season fires. Spring fires occur mainly in the human‐dominated and frequently burned southern edges of the study domain (Figure 1, Figure S1), where roads, burn history, and land cover are important drivers impeding fire growth (Figures 5 and 6), whereas fire weather, surface water, fuel load, and downslope are important for the summer fires that occur in the extensive remote taiga that burns less frequently, is less fragmented, and is dominated by lightning‐ignited fires.

When we zoom into the daily dynamics of the particularly severe fire season of 2020 (Figure S7), we can observe that the importance of changes in fuel moisture, WFSI, and the landscape drivers explaining fire stops is highly variable over time and depends strongly on fire weather (Figure S7a). As the fire season was ramping up in the middle of June, the relative contribution of fuel moisture to explaining fire stops was negligible (less than 10%, Figure S7). However, as the weather changed in early July, VPD and daily burned area declined, and the relative contribution of fuel moisture surged to explain approximately 50% of the fire stops, mainly at the cost of the combined landscape drivers (Figure S7). This oscillating pattern repeated itself multiple times in the following months, indicating a high temporal variability in the relative importance of fire weather and landscape drivers.

We found clear shifts in the time series of the meteorological drivers limiting fire growth around the last recorded burn day at the perimeter (Figure S8). This is to be expected because we use these shifts in the time series to determine whether a fire was stopped by a change in the weather (Section 2.3). Nonetheless, it is still interesting to examine the temporal dynamics and the absolute range of these different drivers. Mean VPD at the fire stops attributed to a decline in VPD was notably higher in the 4 days preceding and lower in the 3 days following the last recorded burn day, compared to the other fire stops (Figure S8a). The median of daily maximum VPD at these fire stops declined from 1.47 kPa at the last recorded burn day to 0.76 kPa the next day (Figure S8a), a decline of 48% and well below the 1.2 kPa empirically derived VPD threshold for fire activity in boreal ecosystems (Balch et al. 2022; Clarke et al. 2022). Changes in surface soil moisture were more subtle, but the difference in surface soil moisture between the fire stops attributed to soil moisture, and the other fire stops were still visible up to 5 days after the last burn day (Figure S8b). Also, the WFSI showed a more subtle decline in the 2 days after the last burn day at the fire stops attributed to WFSI (Figure S8c).

### 3.5 Decadal Trends in the Importance of Drivers Impeding Fire Growth

**FIGURE 8:**
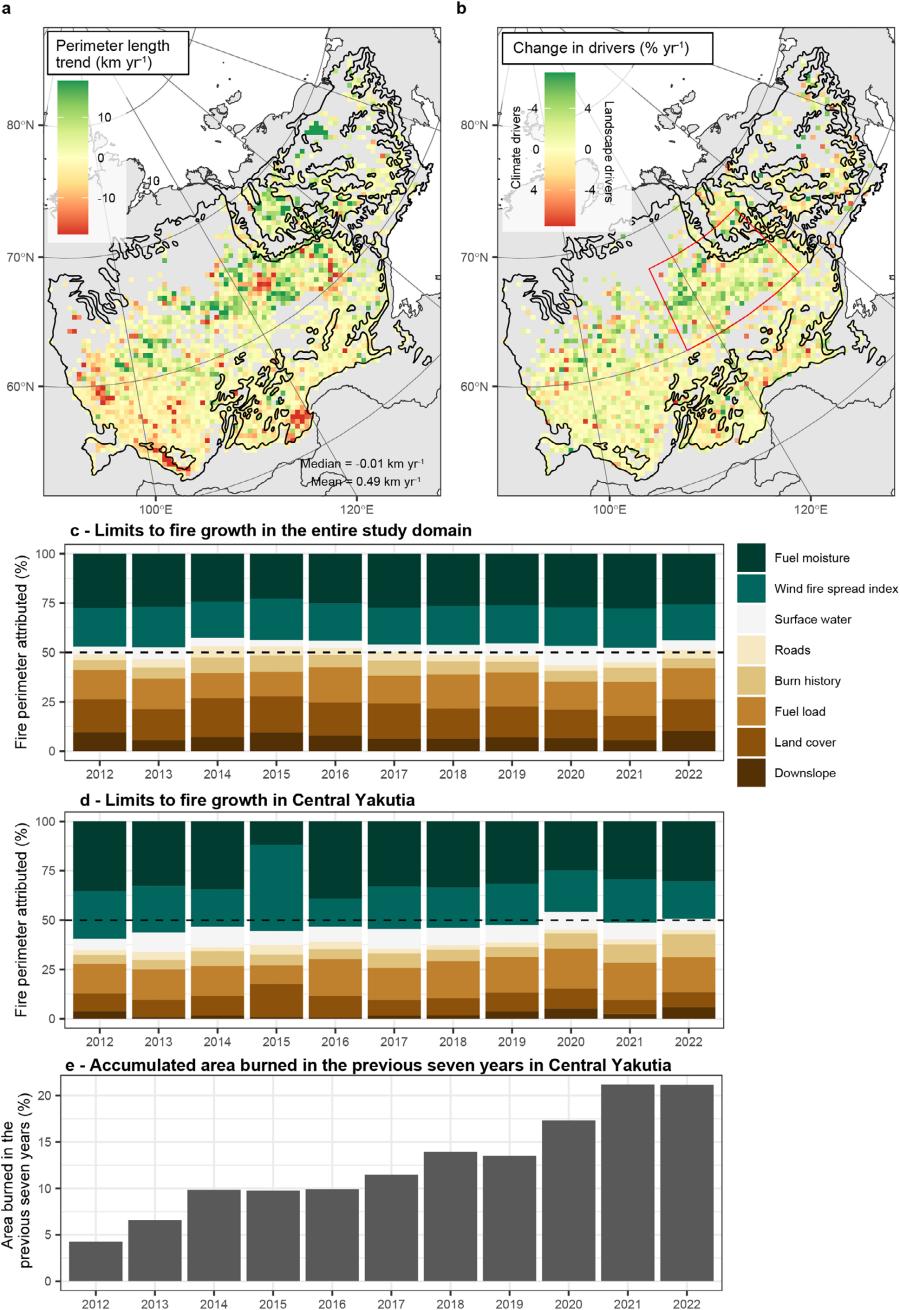
Long‐term trends in perimeter length and the contribution of top‐down climate and bottom‐up landscape drivers to fire cessation (2012–2022). (a) The change in total perimeter length, (b) the change in the contribution of climate versus landscape drivers explaining fire stops, (c) the relative contribution of different drivers expressed as the percentage of the total perimeter explained in the entire study domain, and (d) the relative contribution of different drivers expressed as the percentage of the total perimeter explained in central Yakutia, and (e) the accumulated burned area in the previous 7 years in central Yakutia. The trend maps (a, b) were created using ordinary least squares regression on annually averaged results that were spatially aggregated to a 40 km resolution grid using a Lambert azimuthal equal‐area projection. The central Yakutia sub‐region is indicated as a red polygon in panel (b). Map lines delineate study areas and do not necessarily depict accepted national boundaries.

We found a general increasing trend in the fire perimeter length that has been recorded each year since 2012 (OLS regression, see Section 2.3), mainly in the northern regions (> 60° N) of our study domain (Figure 8a). In these northern regions, the contribution of the combined landscape drivers in explaining fire stops has also increased between 2012 and 2022, at the expense of the combined fire weather drivers (Figure 8b). However, when considering the entire study domain, the relative importance of the eight examined potential drivers impeding fire growth has been relatively stable since 2012 (Figure 8c). There were no statistically significant (*p* < 0.01) trends for a single driver over time for the entire study domain.

The most notable changes in the relative importance of the different drivers occurred in the central Yakutia region (Figure 8b). We delineated this sub‐region (115° W, 135° W, 60° N, 67° N) to examine the temporal trends in the relative contribution of the different drivers impeding fire growth in this recent wildfire hotspot (Figure 8b,d). In this region, the combined contribution of all landscape drivers has significantly increased from 2012 to 2022 (*p* < 0.01, slope = 0.9% year^−1^, *R* ^2^ = 0.68, Figure S9h). Conversely, the combined contribution of the two fire‐weather drivers showed a negative trend, although individually not statistically significant (Figure 8d, Figure S9a,b). The main drivers of the increased contribution of landscape limitations on fire growth in central Yakutia were the increased importance of burn history (*p* < 0.01, slope = 0.51% year^−1^, *R* ^2^ = 0.54, Figure S9d) and fuel load (*p* < 0.05, slope = 0.54% year^−1^, *R* ^2^ = 0.37, Figure S9e). These trends are likely related to the total burned area in the previous 7 years in central Yakutia, which also showed a strong increase from 2012 to 2022 (*p* < 0.01, slope = 1.6% year^−1^, *R* ^2^ = 0.94, Figure 8e). At 50% of all fire stops that were attributed to burn history, a recent fire had occurred less than 6 years ago (Figure S10). This suggests that our method mainly captures relatively short‐term fuel reduction due to recent fires and that these short‐term limits on fuel availability might be important limitations to fire growth in our study domain.

## 4 Discussion

### 4.1 Fire Weather and Landscape Drivers Limiting Fire Growth

We have monitored the development of individual fire perimeters on a daily time step across the vast taiga of East Siberia (5.9 million km^2^). Our method attributed one or more causes of fire cessation to a large majority (87%) of the 2.5 million fire stops analyzed. Almost half (45%) of the explained fire stops were attributed to both a change in the landscape at the fire perimeter as well as a change in fire weather at the time of fire cessation. Roughly, one third of the explained fire stops (32%) were solely explained by the landscape drivers, while the fire‐weather drivers alone accounted for the remaining 23%. This high proportion of fire stops explained by both landscape and fire‐weather drivers suggests that interactions between bottom‐up landscape and top‐down meteorological drivers often determine fire cessation in boreal forests (Cavard et al. 2015; Holsinger et al. 2016; Taylor and Skinner 2003). Furthermore, this highlights a significant future risk that historically effective bottom‐up fire stops may fail under increasingly severe fire weather with climate change (Whitman et al. 2024).

We observed large geographic trends in the importance of top‐down fire‐weather drivers versus bottom‐up landscape drivers limiting fire growth (Figure 5). In the remote north Siberian taiga, fire growth is mainly constrained by fire‐weather drivers, fuel moisture, and wind (Figure 5). Therefore, prolonged warm and dry conditions may not only increase the probability of a fire being ignited by lightning (e.g., Hessilt et al. 2022) but also alleviate the constraints of climatic conditions for fire spread (Figure S7). The results suggest that the highest potential for further fire growth with a warming and drying climate is in the north of the Siberian taiga and less so in the south. This also confirms recent findings that anomalously warm and dry conditions in the north Siberian taiga are strongly linked to extreme fire years (2019, 2020 and 2021) in this region (Kim et al. 2020; Scholten et al. 2022).

A change towards less favorable fire weather for fire spread, either by an increase in fuel moisture or a decline in the WFSI, was the most important driver limiting fire growth in our study domain (Figure 5). A sudden increase in fuel moisture could be attributed to 41% of the fire stops each year (Figure 5), highlighting the importance of VPD driving fire spread and extinction (Balch et al. 2022; Ray et al. 2005; Sedano and Randerson 2014). As fuel moisture is highly dynamic over time, we also found that the contribution of fuel moisture to explaining fire cessation changed dramatically over time, from explaining less than 10% of the fire stops when the fire weather is conducive to fire spread to explaining around 50% a few weeks later as fuel moisture increased again (Figure S7). At perimeter locations where a decline in fuel moisture was observed, the median daily maximum VPD dropped from approximately 1.5 kPa on the last recorded burn day to about 0.8 kPa the following day (Figure S8a). This supports the presence of a VPD threshold in this range, below which boreal fire activity ceases (Balch et al. 2022; Clarke et al. 2022). Our results suggest that a large potential for further fire growth with climate change as warming and drying will increase days with anomalously high VPD not experienced before in these regions (Balch et al. 2022; Sedano and Randerson 2014).

### 4.2 The Importance of Quasi‐Static Landscape Drivers Impeding Fire Growth

We found a relatively small contribution of quasi‐static landscape drivers limiting fire growth in East Siberia, with roads, water bodies, and downslope together explaining only 24% of the fire stops. Roads are often coined as a classic example of fire barriers, limiting burned area and fire size globally (Haas et al. 2022; Narayanaraj and Wimberly 2011). However, road density is low in eastern Siberia (Figure S1b), and therefore, our estimate of roads explaining 5% of the fire perimeter is at the lower end of the range of values found in the literature, with roads explaining 25.7% of the perimeter in the southwest of the United States (Yocom et al. 2019), 15.9% in the Irkutsk Region in eastern Siberia (Kuklina et al. 2023), and 9.6% in the Australian savanna (Fisher et al. 2022). From north to south, with increasing road density, the importance of roads as fire barriers logically increases, which adds to landscape fragmentation and constrains fuel continuity, limiting the growth of fires in southern Siberia and globally (Haas et al. 2022; Macauley et al. 2022).

Our results show that the importance of water bodies acting as fire breaks in eastern Siberia was generally low (7%, Figure 5) but highly dependent on the landscape. Although comparable quantitative attribution studies do not exist, other studies have found no effect of water bodies on fire activity and fire growth in the northwestern United States (Narayanaraj and Wimberly 2011) to substantial effects in the Rocky Mountains (Connor et al. 2017; Macauley et al. 2022; Suffling 1993), the boreal forests of Saskatchewan in Canada (Nielsen et al. 2016), and the Australian savanna (Fisher et al. 2022). Although generally not so important, we found some specific regions in which water bodies were the main landscape constraint on fire growth (Figure 5). Specifically, in the far northeastern part of the study domain, known as the Kolyma Lowland, ice‐rich Yedoma deposits are prevalent and the landscape is dotted with numerous thermokarst lakes and ponds, Here, approximately half of the total fire perimeter was attributed to the presence of surface water (Figure 5, Figure S3). This demonstrates that in landscapes dominated by lakes and other water bodies, these features serve as a major constraint on fire activity and spread (see e.g. Nielsen et al. 2016). The importance of surface water in limiting fire growth in Yedoma permafrost landscapes provides an interesting clue for a potential negative feedback loop as fires are known to initiate permafrost thaw and thermokarst development and therefore create new thaw ponds, lakes, and bogs (Y. Chen et al. 2021; Gibson et al. 2018; Holloway et al. 2020; Jones et al. 2015; Myers‐Smith et al. 2008). Although the processes that drive the development of thermokarst lakes operate on longer timescales (Grosse et al. 2013), more fires in permafrost regions could result in the increased initiation of future thaw lakes, ponds, and bogs, thereby limiting potential future ignitions and constraining fire spread in ice‐rich permafrost landscapes.

The effect of downslope on constraining fire growth was quite important in the mountain tundra ecoregions, explaining 26% of the fire stops (Figure 5). Modeling and experimental studies have shown that downslope generally impedes fire growth due to a lack of fuel preheating and by canceling out wind enhancement effects on fire spread (Eftekharian et al. 2019; Pyne et al. 1996; Sullivan et al. 2014; Weise and Biging 1996). However, while some landscape‐scale studies have examined the effect of terrain slope and terrain ruggedness in general on fire cessation (Connor et al. 2017; Macauley et al. 2022; Narayanaraj and Wimberly 2011), the specific effects of downslope on fire spread or fire cessation have, to our knowledge, not been studied at the landscape scale. Future work should focus on how and to which extent downslope impedes fire growth at the landscape scale and examine the complex interactions between terrain slope and wind dynamics, which can sometimes lead to enhanced fire spread in the downslope direction (Abatzoglou et al. 2023; Anderson 1968).

### 4.3 Spatial and Temporal Trends in Semi‐Dynamic Drivers and Fire Self‐Limitation

The semi‐dynamic landscape drivers limiting fire growth were related to vegetation composition and fuel loading, drivers that can change on decadal time scales depending on the climate, fire regime, and other disturbances. Our results show that these semi‐dynamic drivers, represented by burn history, and especially aboveground fuel load and land cover, are the most important bottom‐up landscape drivers limiting fire growth in eastern Siberia (Figure 5). The contribution of burn history, or the fuel reduction effect due to recent fires, to explaining fire stops was higher in areas dominated by human ignitions that also burn more frequently (14%), compared to natural lightning ignition‐dominated areas (6%, Figure 6a). These estimates are within the range of values found in comparable analyses in the United States, where values of 3% (Teske et al. 2012) to 10% (Belval et al. 2019; Yocom et al. 2019) have been reported, but are much lower compared to the 42% to 96% reported for Portugal (Davim et al. 2021, 2023). Our study in eastern Siberia corroborates observations from other parts of the world (Davim et al. 2021; Holsinger et al. 2016) demonstrating that a higher fire frequency increases the likelihood of fire self‐limitation.

Although of minor importance across the entire study domain, we found a strong increase in the importance of burn history as a driver of fire cessation in the central Yakutia region from 2012 to 2022, a recent fire hotspot that experienced a substantial increase in burning in this period (Figure 8, Figure S9d). While at the start of our fire stop time series in 2012, less than 5% of central Yakutia had burned in the previous 7 years (2005–2011), in 2022, this number had increased to more than 20% (2015–2021). These results suggest that in central Yakutia, a region that experienced record‐breaking fire seasons in recent years (Descals et al. 2022; Romanov et al. 2022; Scholten et al. 2022), fires are becoming increasingly limited by the availability of fuels that were burnt in previous fires and therefore less by climatic constraints. This suggests that the negative feedback mechanism of fire activity on future fire activity via the availability of fuels is already playing out in eastern Siberia, and fire self‐limitation might become more important here in the future (Parks et al. 2016). However, short‐interval reburning might also become more common in periods of severe fire weather that are expected with climate change (Whitman et al. 2024).

We found a stark spatial and temporal contrast in the importance of the two remaining semi‐dynamic drivers limiting fire growth: the aboveground fuel load and land cover (Figure 5). In the southern and southwestern part of the domain, the climate is milder, making permafrost rare and human population densities higher. Fires in this region are therefore mostly ignited by people, and the landscape is more fragmented (Figure 1). Here, the fire encountering a different—presumably less flammable—land cover is the main landscape constraint on fire growth (Figures 5 and 6). This is in contrast to the remote northern taiga (> 60° N) where, due to a colder climate, continuous permafrost soils are present and the fire regime is dominated by lightning ignitions (Figure 1). Here, the aboveground fuel load is the most important landscape constraint on fire growth (Figures 5 and 6). Areas without permafrost generally overlapped with areas with some human ignitions, as well as different land cover types compared to areas with permafrost, which are mainly covered by deciduous needle leaf (*Larix* spp.) forest (Figure 1). Therefore, similar trends are observed when looking at ecoregions, ignition sources, and permafrost cover, such as the difference in the importance of fuel moisture, road networks, and fuel type (Figures 5 and 6). These synchronies in climate, landscape, land cover, and ignition sources might help the parametrization of global fire models (Cardoso et al. 2022; Hantson et al. 2020).

### 4.4 Data Limitations, Improvements, and Outlook

Although we were able to attribute 87% of the fire stops to a significant (*p* < 0.01) change in one or more potential landscape and fire‐weather drivers, we were not able to provide a single potential cause of fire cessation for the remaining 13% of fire stops. We believe that future work can improve on this and map the limitations of fire growth with a higher certainty as well as at a higher spatial resolution. This will, however, require certain current data limitations to be alleviated. First, higher spatial resolution (20–30 m) burned area products are necessary to delineate fires more accurately in space. In our experience, a spatial mismatch between the delineated fire perimeter at 300 m resolution and the landscape variables at 30 m resolution often prevented the accurate attribution of these landscape variables to explaining fire cessation (see e.g., Figure 2g). Furthermore, because it is essential to accurately estimate the time of fire spread cessation, data fusion of high spatial resolution data (e.g., Landsat, Sentinel‐2) with high temporal resolution data (e.g., MODIS and VIIRS) will be essential to improve future perimeter attribution (Boschetti et al. 2015). Finally, future investigations could also incorporate measures of fire severity in order to separate surface‐ and stand‐replacing crown fires (Burrell et al. 2022; Kharuk et al. 2016). These estimates could then be linked to fuel consumption and recovery after fire. These advances in burned area mapping, especially when applied to historical imagery, will also improve the attribution of previously burned area (burn history) and their fuel reduction effects to explaining fire spread cessation.

The second major improvement that could be made is the mapping of fuel load and land cover. In this study, because of data limitations, we used aboveground biomass and percentage tree cover as proxies of aboveground fuel load, while these large trees are only a part of the total fuel load and, in the case of less severe surface fires, are not part of the fuel load at all (Schimmel and Granstrom 1997). Therefore, future work would benefit from high spatiotemporal data that captures sub‐grid scale fuel heterogeneity to accurately predict the growth of boreal fires. Furthermore, as we found that land cover heterogeneity is an important constraint on fire growth (Figure 5c), future analyses would also benefit from new land cover data on a sufficiently high spatial and temporal resolution to capture the effect of land cover on fire spread and the potential feedbacks of fire with land cover changes. Vegetation changes, specifically those driven by fire and climate warming, can trigger alternate successional trajectories from needle leaf to broad leaf dominated forests, which are already observed in both boreal North America (Baltzer et al. 2021; Johnstone et al. 2020) and Siberia (Tautenhahn et al. 2016; Williams et al. 2023). Furthermore, in southern Siberia, repeated fires have also contributed to the degradation of forest to steppe vegetation (Kukavskaya et al. 2016). Therefore, post‐fire succession to less‐flammable broad leaf tree species or forest degradation to steppe vegetation might present other negative feedback loops on fire activity that might constrain future fire growth (Baltzer et al. 2021), which is presently not captured by our method.

Another data limitation that we encountered was the relatively coarse spatial resolution of the climate data (0.1°) derived from the ERA5‐Land product. While ERA5‐Land provides a valuable and widely used source of climate data, its coarse resolution may impact the accuracy of key variables such as wind direction, wind speed, and soil moisture, particularly in topographically complex regions. Reanalysis products, including ERA5‐Land, are known to struggle with accurately resolving surface winds, which may contribute to uncertainties in our analysis (T. Chen et al. 2024; Suo et al. 2024). Although no superior dataset is currently available, these limitations might partly explain the 13% of unknown fire stops observed in our results. Future studies could benefit from higher‐resolution observational data and modeling to improve the representation of these key meteorological variables.

Finally, we did not explicitly account for active human fire suppression as a driver of fire stops, although active fire suppression in eastern Siberia is either selective and focused on populated areas in the south or completely absent (Kharuk et al. 2021). While the remaining 13% of unexplained fire stops in our study could have been partly caused by active fire suppression, we did not find a particular concentration of unexplained fire stops surrounding populated areas. Passive fire suppression in the form of anthropogenic fuel reduction, including through prescribed burning, human‐modified land cover, and roads, was captured by our analysis, and these drivers were indeed more dominant in the populated regions of our study domain (Figure 1, Figure 5c). However, we did not explicitly attribute these fire stops to human activities. Although human‐modified land cover can be a proxy for both active and passive fire suppression (e.g., Scholten et al. 2024), data on human fire suppression, either active or passive, are not available on a continental or global scale. Future work can improve upon our method by explicitly including active fire suppression and attributing landscape drivers to passive suppression, which could provide new insights into anthropogenic and natural fire stops and how these impact fire regimes in boreal forests.

In this study, we examined the drivers of fire cessation across eastern Siberia using nine global datasets and one tailored regional land cover dataset, achieving strong performance across the study domain. While current global land cover products lack the granularity and regional specificity of the tailored land cover dataset that we used, the development of high‐quality pan‐Arctic boreal land cover products could facilitate the broader application of our method across the entire Arctic boreal region and potentially even on a global scale. Although this would pose a significant computational challenge, it would allow for the attribution of fire stops across biomes and at unprecedented spatial and temporal scales globally. Such an effort would greatly enhance the development and parameterization of global fire models, improving fire spread modeling to more accurately represent fire behavior and fire size (Cardoso et al. 2022; Hantson et al. 2020), ultimately reducing uncertainties in future fire regimes and their impacts on human livelihoods and carbon emissions.

## 5 Conclusions

In this study, we have tracked the development of fire perimeters of single fires at a daily time step across the vast East Siberian taiga (5.9 million km^2^) and attributed single or multiple causes of fire cessation at a certain place and time. This allowed us to (a) quantify the importance of different landscape and fire‐weather drivers of fire stops, (b) examine the spatial variability in the importance of these drivers, and (c) explore the temporal dynamics and trends in these drivers from daily to decadal timescales. To our knowledge, this is the first study to examine a multitude of potential drivers of fire cessation in detail, especially over such a large spatial extent. Of the explained fire stops, we attributed 32% to bottom‐up landscape drivers, 23% to top‐down fire weather drivers, and 45% to a combination of both. This high proportion of fire stops (45%) where we found a combined effect of fire weather and landscape drivers suggests that complex interactions between bottom‐up and top‐down variables are often required to effectively impede fire spread in boreal forests (Cavard et al. 2015; Holsinger et al. 2016; Taylor and Skinner 2003). This also suggests that landscape constraints on fire spread, such as fuel load limitations, might become less effective in stopping fires as fire weather becomes more severe with climate change (Abatzoglou et al. 2021; Whitman et al. 2024). Furthermore, our findings reveal distinct emergent fire regimes in eastern Siberia, with fire stops in the remote northern part of our study domain primarily caused by fire weather constraints, above‐ground fuel load, and water bodies, whereas in the southern part of our study domain, landscape fragmentation through land cover transitions, burn history, and roads plays a dominant role in limiting fire spread. Our results provide critical insights for the development and parameterization of global fire models, enabling future modelling efforts to more accurately represent the factors that constrain fire growth in boreal forests. By incorporating the complex interplay between landscape limitations and increasingly severe fire weather driven by climate change, these models will be able to better predict fire behaviour and its impacts under future conditions.

## Author Contributions

**Thomas A. J. Janssen:** conceptualization, data curation, formal analysis, investigation, methodology, visualization, writing – original draft. **Sander Veraverbeke:** conceptualization, funding acquisition, project administration, supervision, writing – review and editing.

## Conflicts of Interest

The authors declare no conflicts of interest.

## Supporting information


Data S1.

## Data Availability

The data and code that support the findings of this study are openly available in Zenodo at https://doi.org/10.5281/zenodo.14967055 and https://doi.org/10.5281/zenodo.14964728, respectively. The code can also be accessed from GitHub at https://github.com/ThomasJanssen90/FireLimits. The ERA5‐Land observational data were obtained from the Copernicus Climate Change Service (C3S) Climate Data Store (CDS) at https://doi.org/10.24381/cds.e2161bac. The burned area data from C3S and the European Space Agency Climate Change Initiative (ESA‐CCI) were also obtained from the C3S CDS at https://doi.org/10.24381/cds.f333cf85. ESA‐CCI above‐ground biomass data was obtained from the CEDA archive at https://doi.org/10.5285/02e1b18071ad45a19b4d3e8adafa2817. Percentage tree cover data was retrieved from the Global Forest Change 2000–2023 dataset available at https://storage.googleapis.com/earthenginepartners‐hansen/GFC‐2023‐v1.11/download.html. Surface water wat obtained from the Global Surface Water dataset (1984–2021), available at https://global‐surface‐water.appspot.com/download. Permafrost zonation was obtained from the Ground Temperature Map, 2000–2016, Northern Hemisphere Permafrost dataset at https://doi.org/10.1594/PANGAEA.888600. Yedoma presence was obtained from the Database of Ice‐Rich Yedoma Permafrost at https://doi.org/10.1594/PANGAEA.861733. Ecoregions were obtained from the Terrestrial Ecosystems of the World (TEOW) from WWF‐US (Olson) dataset at https://www.sciencebase.gov/catalog/item/508fece8e4b0a1b43c29ca22.
