# Supplementary material for: What Are the Limits to the Growth of Boreal Fires?
Source: Glob Chang Biol. 2025 Mar 18;31(3):e70130. doi: 10.1111/gcb.70130 (PMC11915198; doi:10.1111/gcb.70130)
Supplement: Supplementary file 1 — Data S1. [file GCB-31-e70130-s001.docx]

Supporting information

What are the limits to the growth of boreal fires?

Thomas A. J. Janssen_1,2_* and Sander Veraverbeke_2,3_

* corresponding author

_1_ Plant Ecology and Nature Conservation Group, Wageningen University & Research, Wageningen, The Netherlands

_2_ Faculty of Science, Vrije Universiteit Amsterdam, Amsterdam, the Netherlands

_3_ School of Environmental Sciences, University of East Anglia, Norwich, United Kingdom

## Supplementary Methods – Data used

### Burned area and active fires

To delineate the fire perimeters, we harmonized three different burned area and active fire datasets. First, we obtained the European Space Agency (ESA) Fire Climate Change Initiative FireCCI51 250m burned area product, available from 2001 to 2020 (Lizundia-Loiola et al., 2020). The FireCCI51 product is based on satellite data from the Moderate Resolution Imaging Spectroradiometer (MODIS). The second burned area product we obtained was from the Copernicus Climate Change Service (C3S), which is derived from observations of the Ocean and Land Color Instrument (OLCI) on board the Sentinel-3 satellite that are available since 2017 (Lizundia-Loiola et al., 2021). The data were retrieved from the Copernicus Climate Data Store at a 300-meter spatial resolution from 2017 to 2022. Finally, we obtained the 375 m resolution Visible Infrared Imaging Radiometer Suite (VIIRS) active fire dataset, VNP14IMGML, from 2012 to 2022 (Schroeder & Giglio, 2018).

The three datasets were harmonized to obtain a single burned area dataset for the period between 2012 and 2022. The 300 m resolution grid of the C3S dataset was used as the standard grid. The VIIRS active fire dataset, which includes timing and location of active fires, was projected on this same grid. When burned pixels overlapped between the VIIRS data and the two burned area datasets, the burn date of the active fire product was used as these can be considered the most accurate burn time estimates, especially since the FireCCI product is known to have a relatively poor temporal accuracy (Katagis & Gitas, 2022). In this way, all pixels with a fire recorded in one of the three datasets were maintained to minimize omission errors. For our purpose of delineating fire perimeters, commission errors were deemed less problematic because this would only result in a larger perimeter. There were three periods of partial overlap in the fire datasets, from 2012 to 2016 we used the VIIRS data in combination with the FireCCI data, from 2017 to 2020 we used all three datasets and in the last two years (2021 and 2022) we only used the C3S and VIIRS datasets due to the discontinuation of the FireCCI dataset after 2020. After harmonizing the three burned area and active fire datasets, single pixels without a burn date in between detected fires were filled using the average burn date of adjacent pixels, only if two or more of the adjacent pixels had a burn date value, following first order Queen’s adjacency.

### Fire variables derived from burned area

From the harmonized burned area data, we obtained various fire variables that were used in the subsequent analyses. First, we calculated the direction of fire spread in degrees. This was done by smoothing the burn dates with a focal filter using a 11 by 11 pixels window. We then calculated the inverse of the burn dates so that the pixels with the lowest burn dates, representing the start of the fire, became local maxima, while the latest burn dates on the edges of the fire became local minima. Subsequently, we computed the aspect of the inverse burn dates, representing the direction of maximum change in burn dates using the terrain function from the R package *terra*.

### Fire weather variables

The assess the contribution of changes in fire weather to halting fire spread, we obtained three weather variables from the 5^th^ generation European Centre for Medium-Range Weather Forecasts (ECMWF) reanalysis land product (ERA5-Land, Muñoz Sabater, 2019) from 2012 to 2022, that are considered important for fire spread. We obtained hourly air temperature and dewpoint temperature at 2 meter above the surface at a 0.1-degree spatial resolution that were used to calculate hourly vapor pressure deficit (VPD) in kPa using Tetens equation following Monteith and Unsworth (2013). VPD is the difference between the saturated vapor pressure, which increases non-linearly with air temperature, and the actual vapor pressure. VPD is indicative of air aridity, drives surface fine fuel moisture content, and has been positively related to ignition efficiency and fire spread (e.g. Sedano & Randerson, 2014). Also, the surface volumetric soil moisture content (0-7 cm) was obtained from the ERA5-Land dataset at the same temporal and spatial resolution. In contrast to the VPD, the surface soil moisture content shows slower temporal dynamics and is expected to be indicative of general fuel dryness or wetness, including both smaller and larger surface fuels as well as standing fuels.

The final weather variable used in the analysis was the wind fire spread index (WFSI) that we developed for our study. The WFSI uses hourly wind direction and wind speed data from ERA5-Land:

$$Wind fire spread index= \left\{ \begin{aligned} v_{w}\left( 0.5-\sin\left( \frac{2\pi-\sqrt{\left( \theta_{w}-\theta_{f} \right)^{2}}}{2} \right) \right) if \sqrt{\left( \theta_{w}-\theta_{f} \right)^{2}> \pi} \\ v_{w}\left( 0.5-\sin\left( \frac{\sqrt{\left( \theta_{w}-\theta_{f} \right)^{2}}}{2} \right) \right) if \sqrt{\left( \theta_{w}-\theta_{f} \right)^{2}\leq\pi} \end{aligned} \right.$$

where $v_{w}$ is the wind velocity at 2 meter (m s^-1^) and $\theta_{f}$ and $\theta_{w}$ are the dominant direction of the fire front and the wind direction in radians. The second part of the equation provides a value in between -0.5, indicating that the wind direction is opposite to the direction of the fire front, and 0.5, which indicates that the wind direction is perfectly aligned with the direction of the fire front. Multiplying the wind speed with this factor results in that strong winds aligned to the direction of the fire give relatively high positive index values, whereas strong winds opposite to the fire front give low negative values. A decline in the WFSI around the time the fire stopped at the eventual perimeter suggests that the wind direction and speed changed unfavourably for the fire to spread in that direction, effectively halting fire spread.

### Aboveground biomass and percentage tree cover

We obtained aboveground biomass (AGB) as a proxy of fuel load from the European Space Agency’s Climate Change Initiative (ESA CCI) Biomass project (Santoro et al., 2021). The most recent version of the dataset, version 4, was retrieved from ESA’s Climate Data Dashboard, which included the years 2010 and 2017-2020. The AGB data in Mg ha^-1^ was available on a global scale and a 100 m spatial resolution. The AGB for missing years (2011-2016) was estimated using a simple linear interpolation between the observations in 2010 and 2017.

In addition, we obtained percentage tree cover at a 30 m spatial resolution from the Global Forest Change 2000-2022 dataset (Hansen et al., 2013). This dataset includes, among others, a percentage tree cover map for the year 2000 and a map indicating the year of observed forest loss. To retrieve an estimate of the percentage tree cover in each year, we simply set the percentage tree cover values for the year 2000 to zero iteratively for each year a loss was observed. This method does not consider forest regeneration or growth and assumes a total loss of tree cover when a loss has been detected. Data limitations prevented us to make a more realistic annual estimates of tree cover at this high spatial resolution.

### Burn history

For each year of the analysis (2012-2022) we created a gridded dataset estimating the fuel reduction due to burn history. Since most fires in the Eurasian boreal region are low-intensity surface fires (Rogers et al., 2015) the recovery of fuels can be relatively fast. Depending on the dominant tree species and fire severity, the limited literature from our study domain indicates a full recovery of the understory fuel load within 7 to 15 years after a surface fire (Kukavskaya et al., 2014, 2023). We obtained the fuel recovery data from Kukavskaya et al. (2023) for the available dominant tree species in our study domain: Siberian pine (*Pinus sibirica*), Scots pine (*Pinus sylvestris*) and Siberian larch (*Larix sibirica*). We inverted the fuel recovery measurements to obtain the relative fuel reduction as a function of time since fire (Fig. S2). We then fitted a simple exponential function to the data, which provided us with an estimate of the reduction of fuel loading in the years since fire:

$RFR=0.65e^{-0.19t}$

where $RFR$ is the relative reduction of fuel loading compared to pre-fire fuel loading and $t$ is time since last fire in years. We used the FireCCI burned area data for the period between 2001 and 2011 and the harmonized burned area data between 2012 and 2022 to calculate the time since last fire for all pixels and for each year of the analysis (2012-2022). All pixels without a recorded fire were set to 0, meaning no effect of burn history on fuel reduction.

### Land cover types and ecoregions

To account for spatial changes in land cover, we harmonized a forest type and a land cover type dataset for the Russian federation. We preferred these country-specific maps that were created with a focus on the specific vegetation and land cover of Russia over global land cover maps that sometimes fail to capture important local distinctions in land cover. First, we obtained the forest cover map of Russia for the year 2010 developed in the framework of the Proba-V-TerraNorte project. The forest cover map was derived from harmonizing MODIS spectral bands of different spatial resolutions (at about 230m and 460m resolution over Russia) to obtain a forest cover map at a relatively high spatial resolution of 345 meter (Bartalev et al., 2010). This dataset includes detailed distinctions between different forest types but does not include land cover types other than forest. To obtain the land cover types that are not forest, we filled the missing values in the forest cover product with land cover types from the 1 km spatial resolution land cover map of Eurasia for the year 1999, developed under the umbrella of the European Commission’s Joint Research Center and the Russian Academy of Sciences (Bartalev et al., 2003). We merged similar classes in the two datasets and derived a harmonized land cover map including 25 land cover classes from which five are different forest classes (Fig. 1b).

To delineate our exact study area and analyse differences in fire spread limitations between different regions we used the “Terrestrial Ecoregions of the World” map from the World Wide Fund for Nature (Olson et al., 2001). Ecoregions are a way to classify the Earth's terrestrial environments into distinct, ecologically significant regions based on their environmental and ecological characteristics (Fig. 1a).

### Elevation

We obtained terrain elevation from the Japan Aerospace Exploration Agency (JAXA) Advanced Land Observing Satellite-2 (ALOS-2) Phased Array L-band Synthetic Aperture Radar (PALSAR) World 3D digital elevation model version 4.0 (Takaku et al., 2020). This global dataset provides high accuracy elevation above mean sea level (m) at a 30 m spatial resolution using multiple source datasets with a relatively long acquisition period of 12 years between 2006 and 2017 (Takaku et al., 2020).

### Roads

We integrated three distinct road datasets to enhance data completeness and accuracy. The primary dataset used in this analysis is OpenStreetMap, which is an open source dataset made available by the OpenStreetMap Contributors (2022). To address potential data gaps and enhance the overall road network representation, two supplementary datasets were incorporated into the analysis. These supplementary datasets comprise the Global Roads Open Access Data Set (gROADS), version 1 (CIESIN, 2013) and the Global Roads Inventory Project, version 4 (GRIP4) (Meijer et al., 2018). All three datasets were obtained in a vector format (Fig. S1a), which were subsequently rasterized to the same 30 meter grid of the Hansen et al. (2013) tree cover dataset. Because of uncertainties in the road locations, we created a buffer and identified all pixels directly adjacent to pixels identified as road. The pixels identified as road were given the value 1 (100% road cover), the buffer pixels were given the value 0.5 (50% road cover) and all other pixels were given the value 0 (0% road cover).

### Surface water

The presence of surface water, in the form of rivers, streams and lakes, can present an obvious direct barrier preventing fire spread. For this reason, we obtained the Global Surface Water dataset (Pekel et al., 2016). We used the yearly classification layer, which identifies areas with permanent water, seasonal water, and no water. The data is available on a 30 m resolution, is available from 1984 and comes with annual updates. We obtained the yearly classification datasets for the years 2012 to 2021. As the classification dataset for the year 2022 was not yet available, we used the surface water data of 2021 to attribute the fire perimeters of 2022.

### Permafrost

The presence of permafrost and the different distribution classes of permafrost soils were derived from Obu et al. (2019). To create a full cover map of permafrost distribution and for easier interpretation, we merged the sporadic (between 10 and 50% permafrost cover) and discontinuous (between 50 and 90% permafrost cover) permafrost cover classes and merged the area in our study not included in the dataset of Obu et al. (2019) with the area covered by isolated patches (between 0 and 10% cover). In addition, we included the presence of Yedoma from Strauss et al. (2022), which only occur in the continuous permafrost zone. In this way, we used a permafrost cover map containing four classes: Continuous permafrost cover (more than 90% permafrost cover) including Yedoma, continuous permafrost cover (more than 90% permafrost cover) without Yedoma, sporadic-discontinuous permafrost cover (between 10 and 90% permafrost cover) and isolated patches (between 0 and 10% permafrost cover) of permafrost (Fig. 1c).


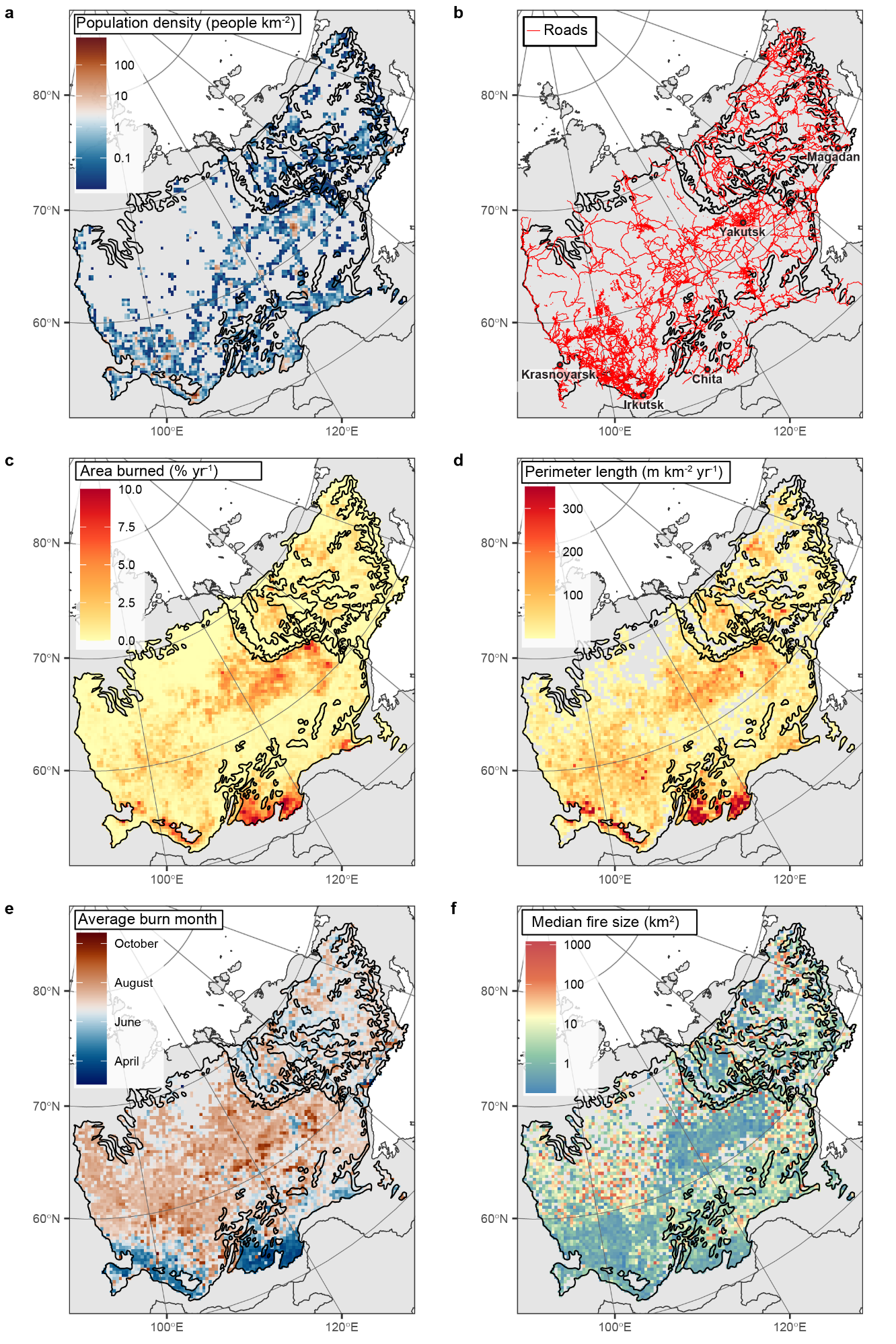


Figure S1 Characteristics of the study area. a) human population density, b) the presence of roads and major cities, c) average percentage land area burned per year, d) average fire perimeter length per unit area per year, e) average month of burning f) median fire size.


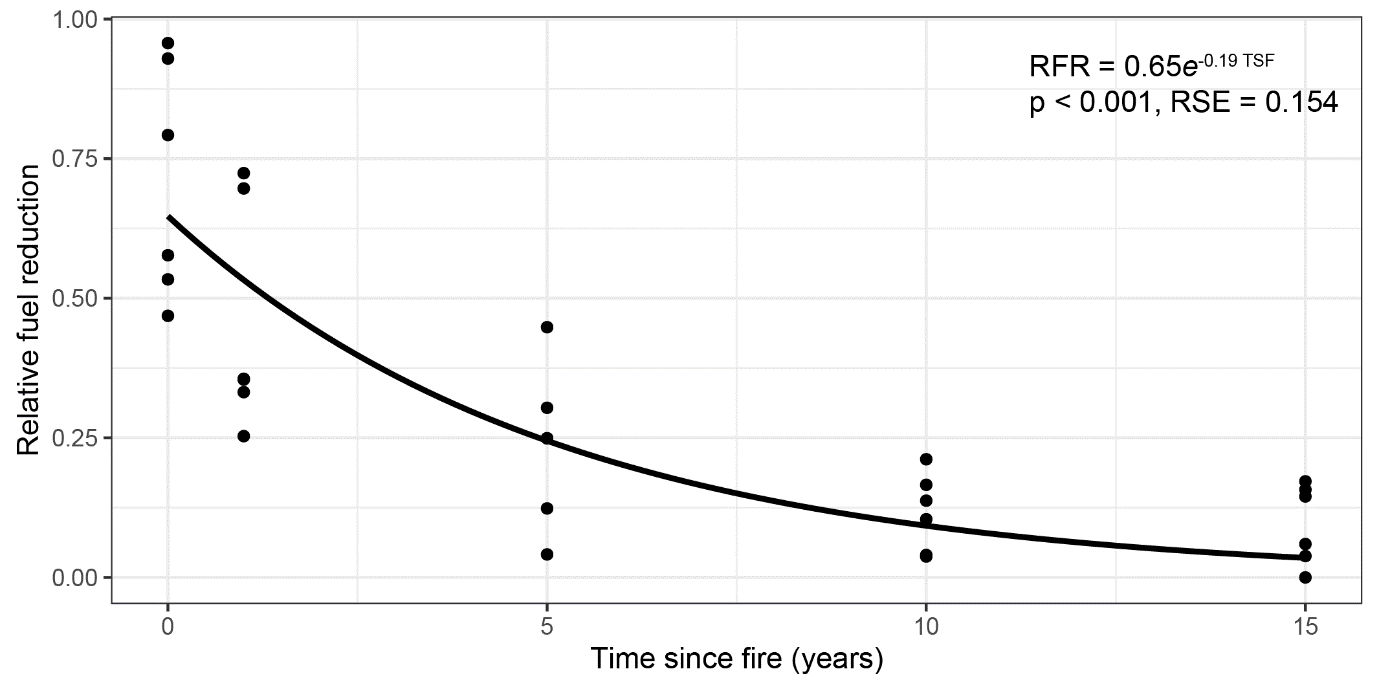


Figure S2 The relationship between relative fuel reduction (RFR) and time since fire (TSF) for Siberian pine (*Pinus sibirica*), Scots pine (*Pinus sylvestris*) and Siberian larch (*Larix sibirica*) stands in eastern Siberia. All data were retrieved from Kukavskaya et al. (2023) and inverted to obtain relative fuel reduction from fuel loading as percentage of pre-fire fuel. The data was scaled by adding the lowest negative value in the data to obtain only positive values for the relative fuel reduction.


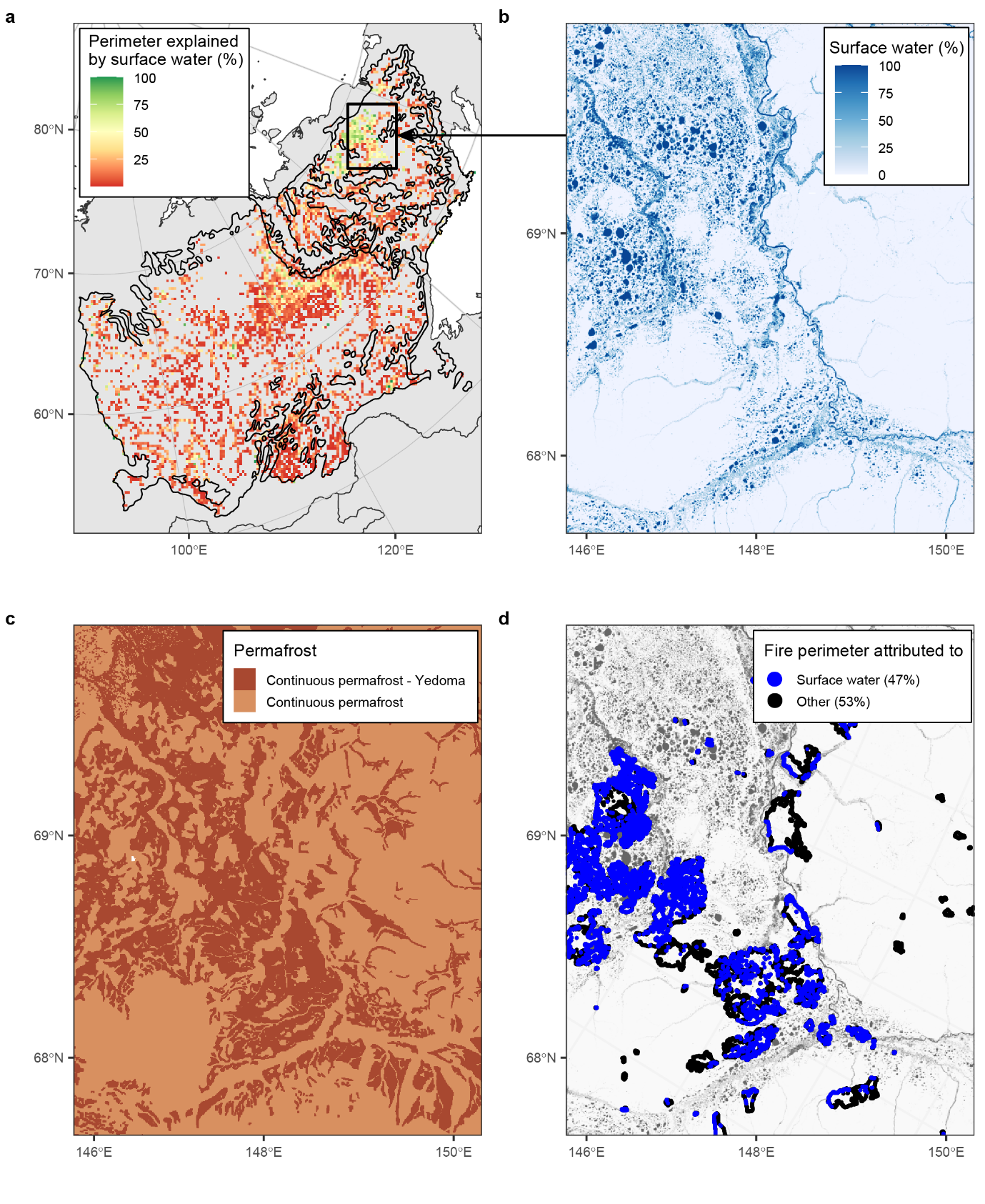


Figure S3 The importance of surface water in limiting fire growth in the Yedoma permafrost regions a) the relative contribution of surface water in explaining fire stops across eastern Siberia (2012-2022), b) the fraction of surface water in the year 2020 in the Kolyma Lowland, northeast Siberia, c) the distribution of continuous permafrost soils with and without Yedoma across the Kolyma Lowland d) fire stops from the severe 2020 fire season attributed to surface water in the Kolyma Lowland. Permafrost distribution is derived from Obu et al. (2019) and Yedoma presence is derived from Strauss et al. (2022). Surface water was derived from the Global Surface Water dataset (Pekel et al., 2016).


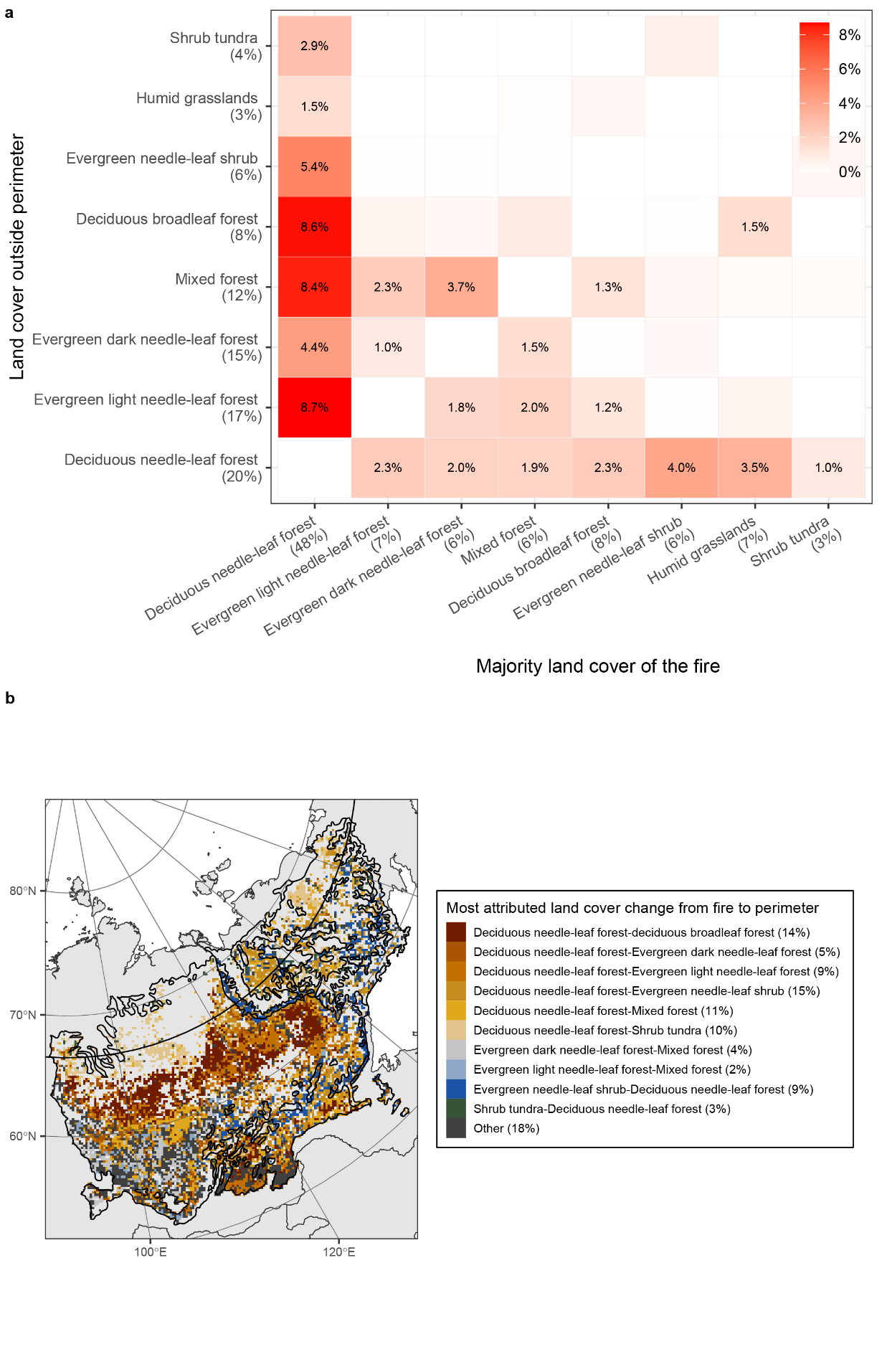


Figure S4 The most frequent changes in land cover along a gradient from inside to outside the fire perimeter that might have contributed to fire cessation. a) the change in land cover from inside to outside the fire perimeter expressed as percentage of fire stops at which a significant (p < 0.01) change in land cover was detected. b) the spatial distribution of the most attributed change in land cover from inside to outside the fire perimeter. Only eight out of a total of 23 land cover classes were depicted that included a change from or to in more than 4% of all fire stops for which a significant change in land cover was detected. Therefore, the percentages on the y and x axis in panel a do not add up to 100%.


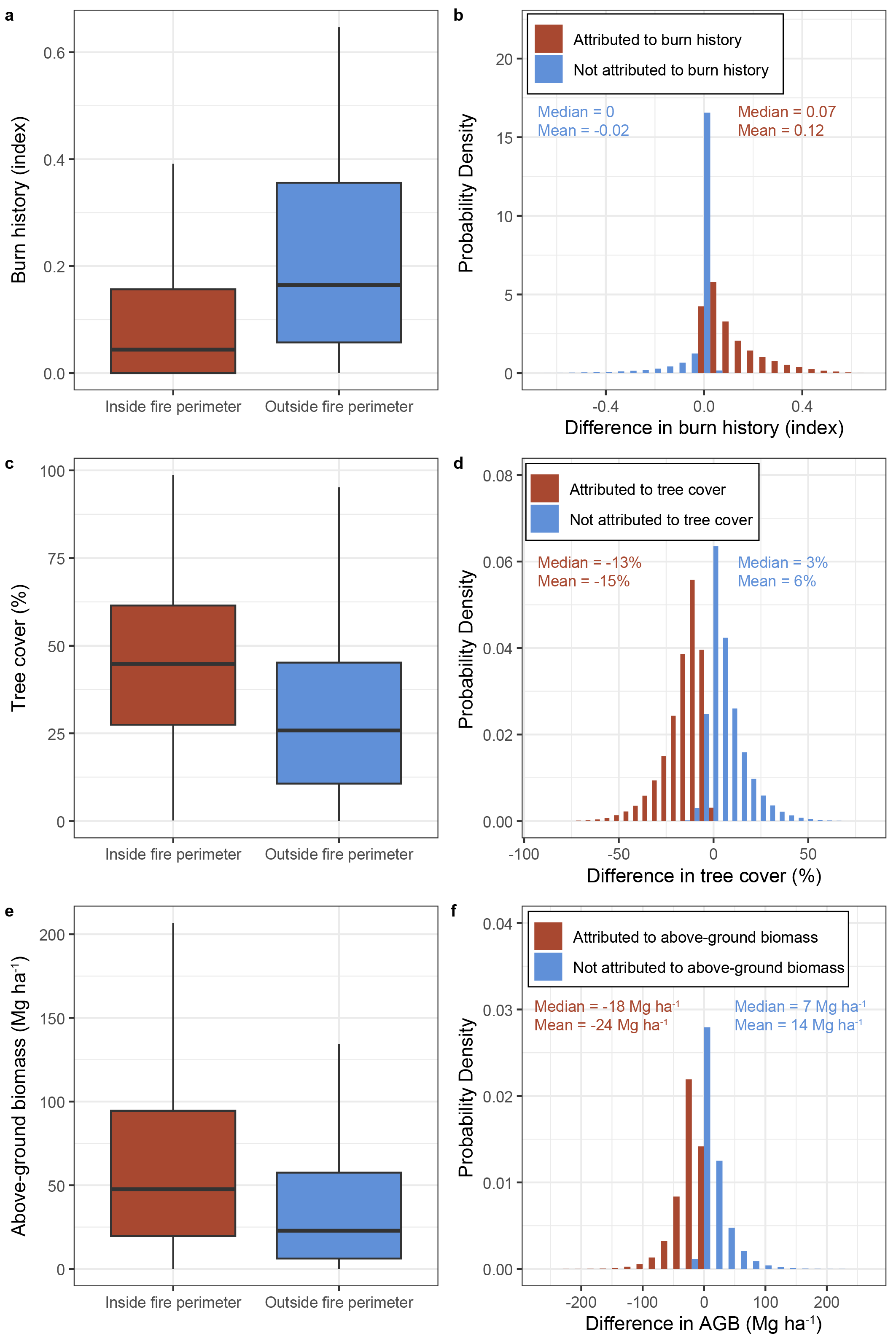


Figure S5 changes in three continuous landscape variables from inside to outside the fire perimeter. The boxplots (a, c, e) show the absolute values of the variables inside and outside the fire perimeter at locations where these variables showed a significant increase (a) or decrease (c, e). The panels containing the histograms (b, d, f) show the probability density of the difference in the variable from inside to outside the perimeter, both for locations where this variable showed a significant difference (p < 0.01), and where it did not. Above-ground biomass = AGB


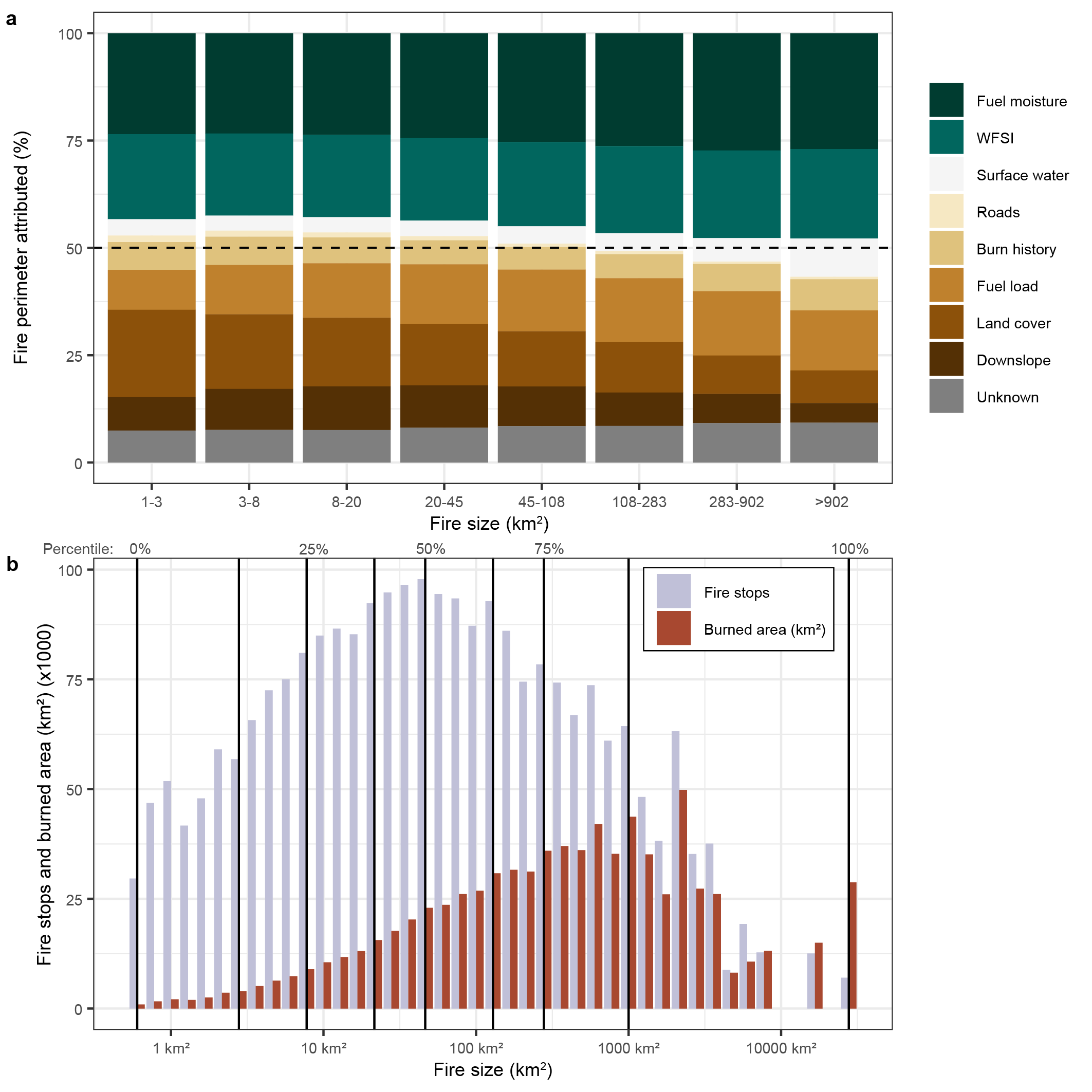


Figure S6 The effect of fire size on the relative importance of drivers limiting fire growth. a) the relative distribution of drivers limiting fire growth grouped into eight fire size groups with equal numbers of fire stops. b) the distributions of fire stops and burned area in relation to fire size. The vertical black lines in panel b) are the percentiles from which the boundaries of the fire size groups in a) are derived.


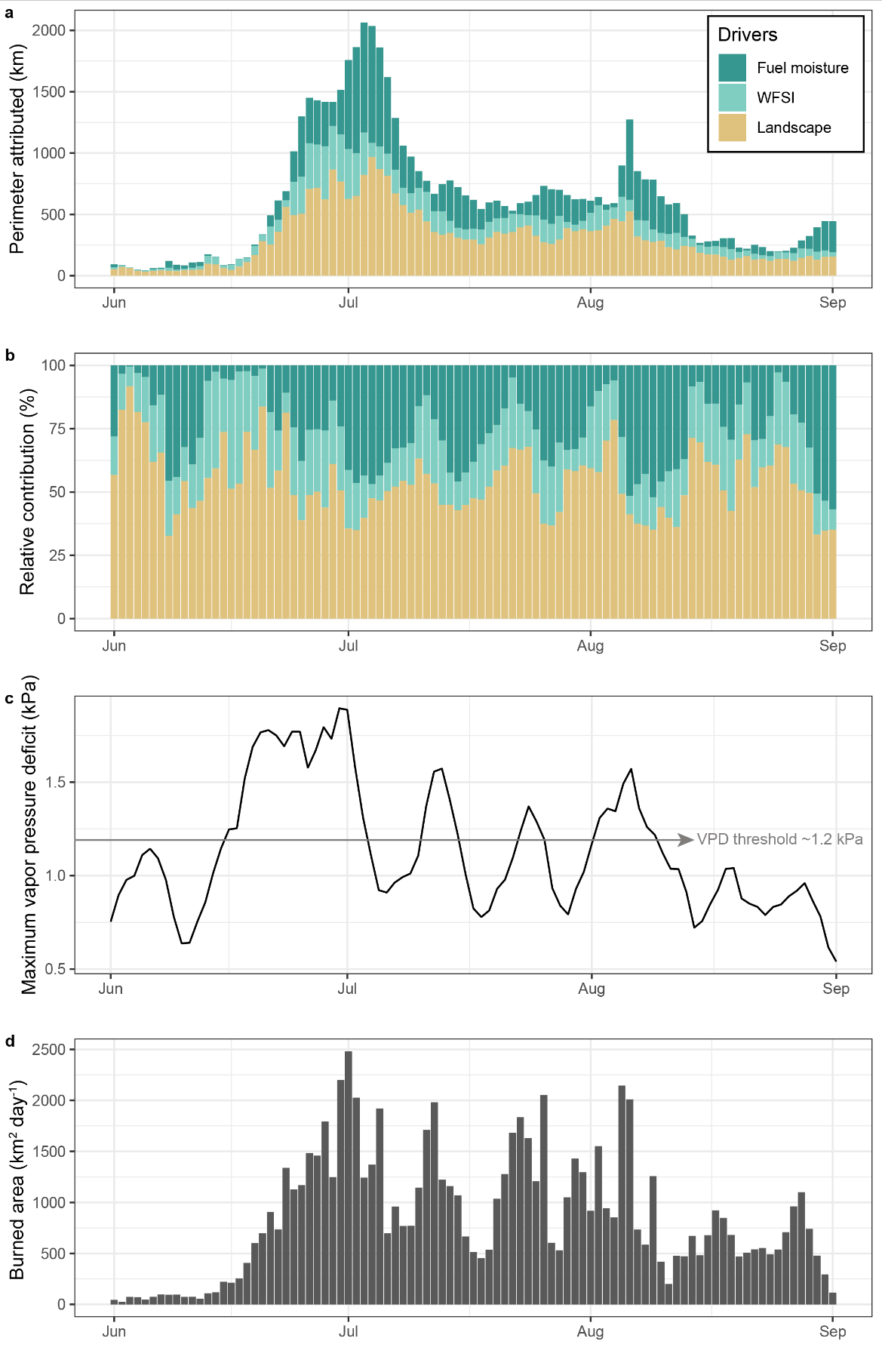


Figure S7 the contribution of the two fire weather drivers and the combined landscape drivers in impeding fire growth during the extreme fire season of 2020. a) the amount of perimeter attributed to temporal changes in fuel moisture -derived from vapor pressure deficit (VPD) and soil moisture- the wind fire spread index (WFSI) and spatial changes in landscape drivers. b) the relative contribution of the different drivers. c) the ERA5-Land derived maximum daily VPD averaged over the entire study domain and d) the daily burned area in the study domain. The grey horizontal arrow indicates the 1.2 kPa VPD threshold below which fire activity is found to be extremely rare in these boreal ecosystems, derived from Balch et al. (2022) and Clarke et al. (2022).


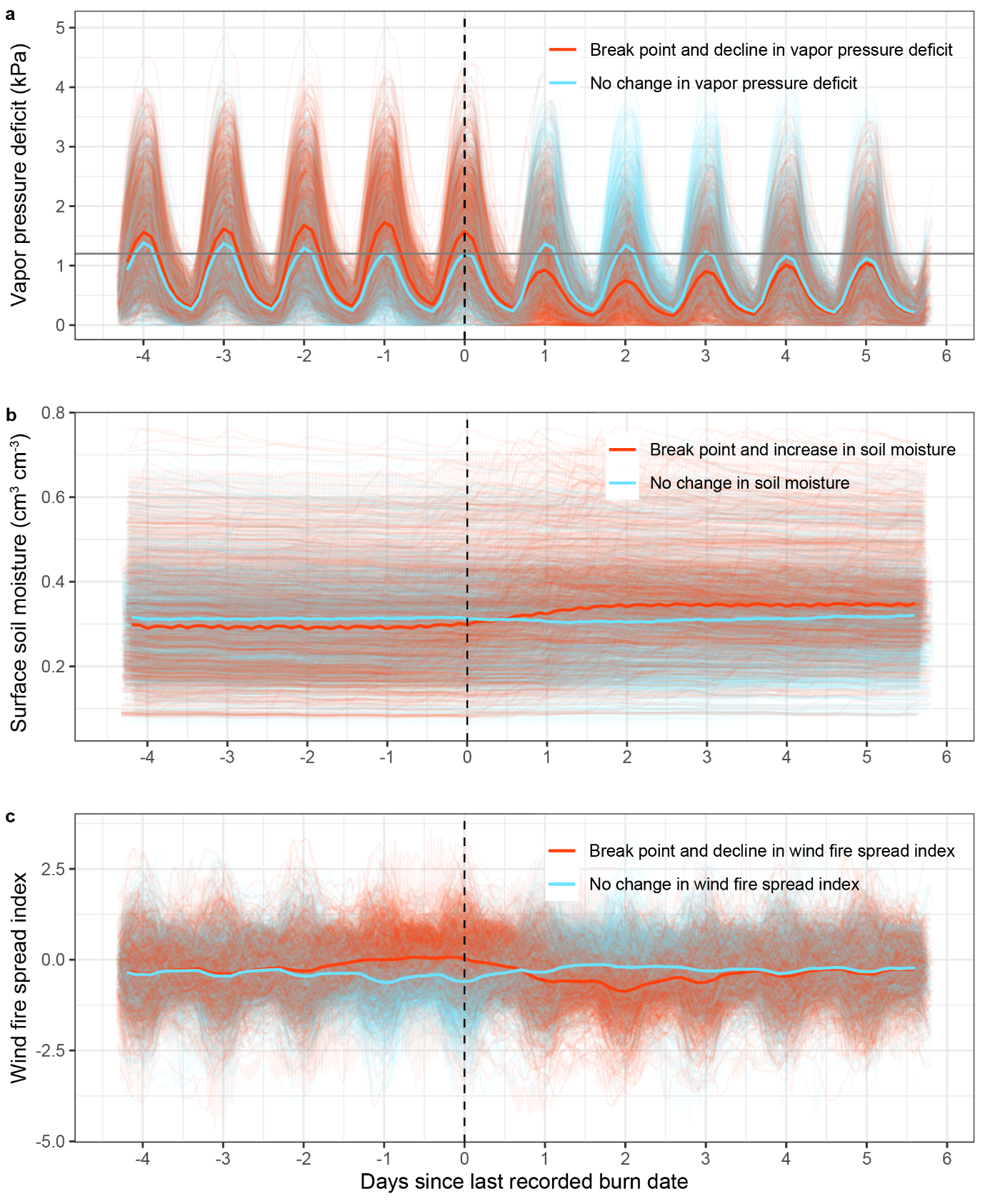


Figure S8 Overview of the fire weather drivers of fire cessation. Time series are for a) vapor pressure deficit (VPD), b) surface soil moisture content (< 9 cm) and c) wind fire spread index. Because of visualization constraints we sampled for each panel 1000 random fire stops where we observed a breakpoint and a significant change in one of the three variables and another 1000 random fire stops where we did not observe such a change. Each location consists of 240 hourly observations (10 days). The time of all locations was changed to local solar time and the x-axis was shifted so that 0 indicates the last day with recorded burning at 16:00 local solar time, which is when VPD peaks. The hourly average of all locations is indicated with a solid line. The grey horizontal line indicates the 1.2 kPa VPD threshold below which fire activity is found to be extremely rare in boreal ecosystems, derived from Balch et al. (2022) and Clarke et al. (2022).


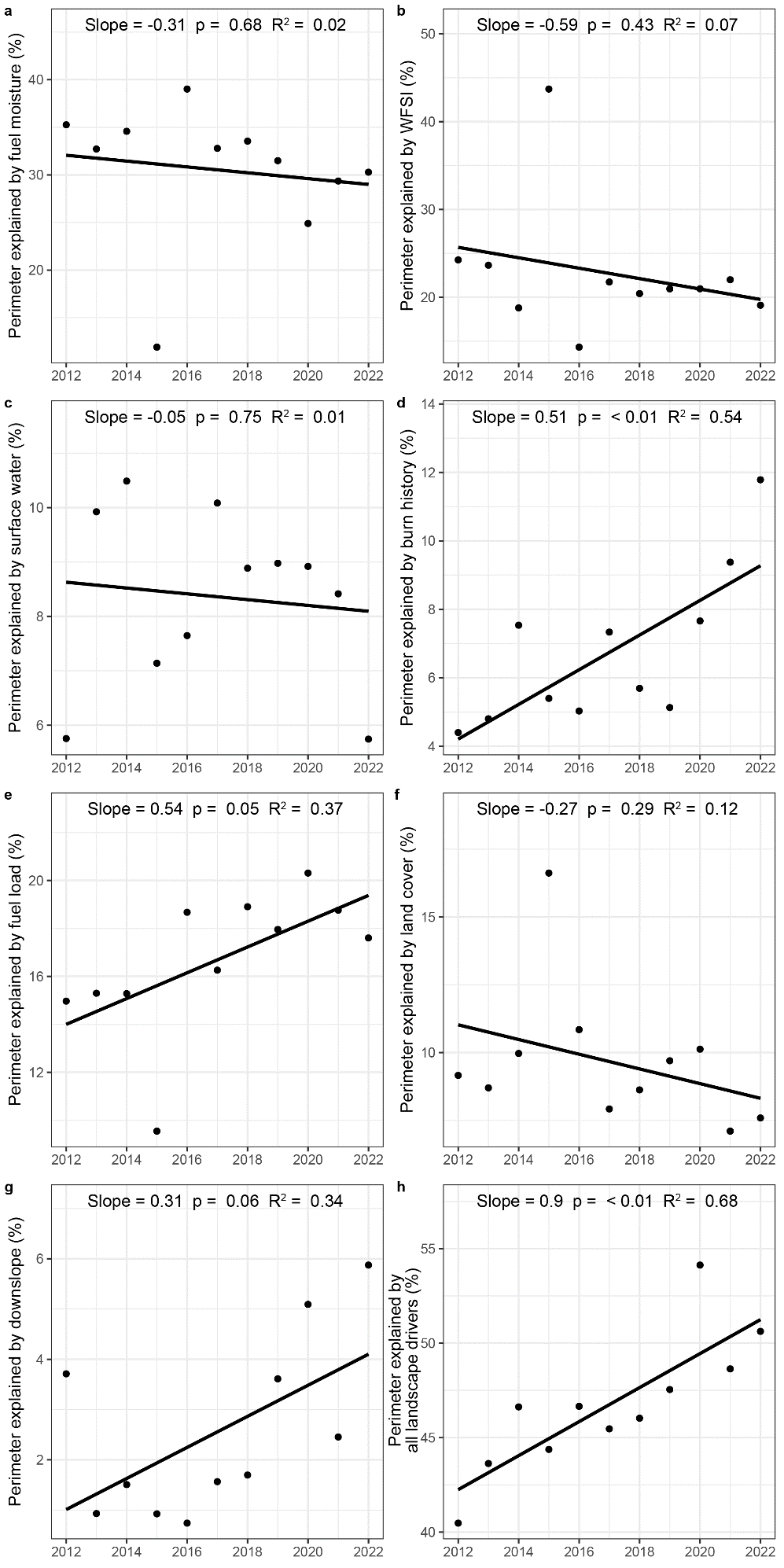


Figure S9 the change in the contribution of different drivers to explaining fire stops in central Yakutia. The included drivers are a) fuel moisture, b) wind fire spread index (WFSI), c) surface water, d) burn history, e) fuel load, f) land cover, g) downslope and h) the combined contribution of all landscape drivers. The sub-region of central Yakutia is indicated with a red polygon in Figure 9b. Note that the slope values reported are in % per year.


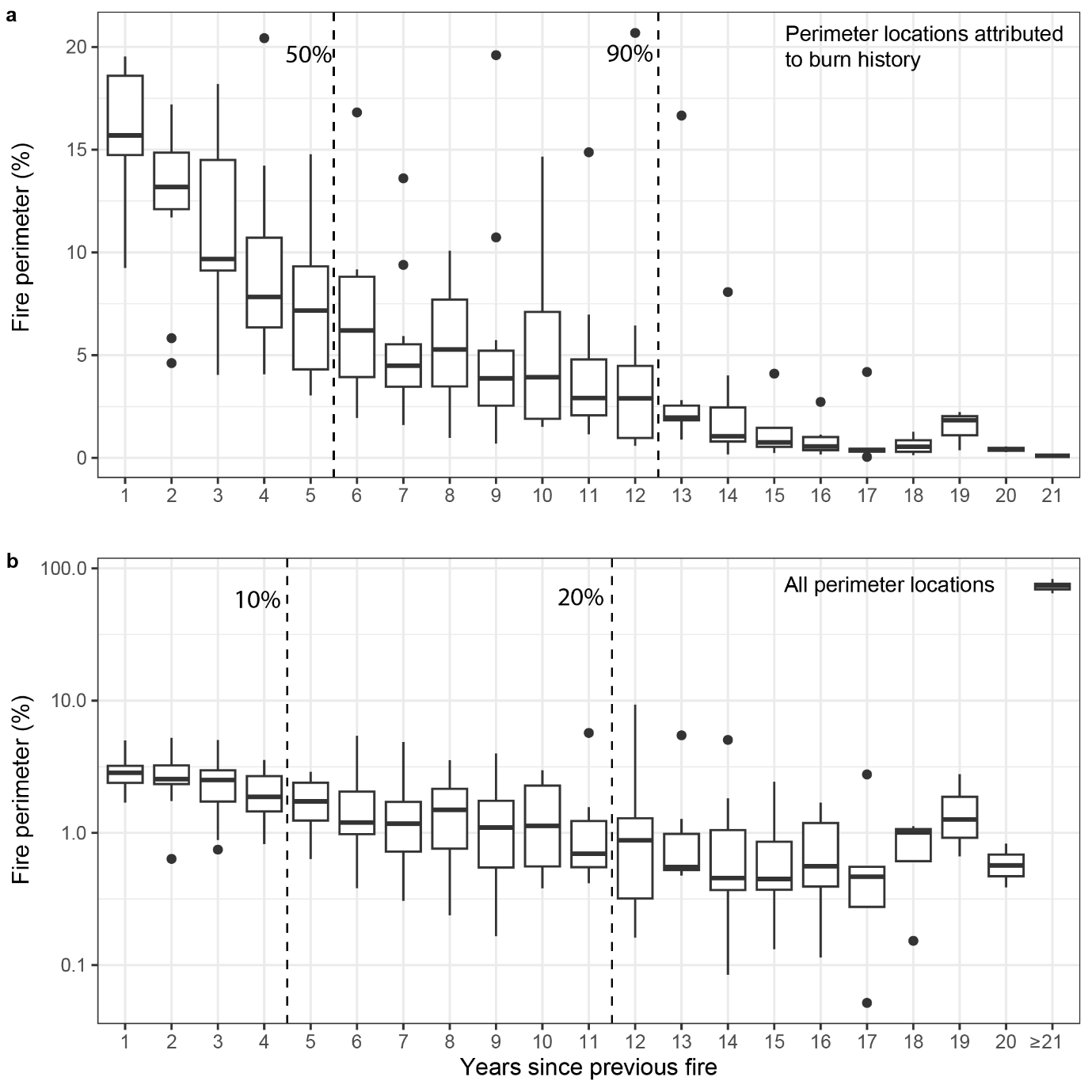


Figure S10 the distribution of time since previous fire for fire stops in northeastern Siberia (2012-2022) with a) the fire stops attributed to being caused by burn history and b) all fire stops. The vertical lines show for panel a) the 50% and 90% quantiles and for panel b) the 10% and 20% quantiles. Note the log scale in panel b. All locations in panel b) with a time since last fire of 21 years or longer, which were not recorded in the satellite record dating back to 2001, are grouped together and represent 73% of the fire stops.

# References

Bartalev, S. A., Belward, A. S., Erchov, D. V., & Isaev, A. S. (2003). A new SPOT4-VEGETATION derived land cover map of Northern Eurasia. *International Journal of Remote Sensing*, *24*(9). https://doi.org/10.1080/0143116031000066297

Bartalev, S. A., Egorov, V., & Kuzmenko, N. (2010). *Forest Cover of Russia*. http://smiswww.iki.rssi.ru/default.aspx?page=356

CIESIN. (2013). Global Roads Open Access Data Set (gROADS), v1. *Online: Http://Sedac.Ciesin.Columbia.Edu/Data/Set/Groads-Global-Roads-Open-Access-v1 (Accessed 02/05/2014)*.

Geofabrik, D. S. (2022). *Open Street Map*. Geofabrik Download Server.

Hansen, M. C., Potapov, P. V., Moore, R., Hancher, M., Turubanova, S. A., Tyukavina, A., Thau, D., Stehman, S. V., Goetz, S. J., Loveland, T. R., Kommareddy, A., Egorov, A., Chini, L., Justice, C. O., & Townshend, J. R. G. (2013). High-resolution global maps of 21st-century forest cover change. *Science*, *342*(6160), 850–853. https://doi.org/10.1126/science.1244693

Katagis, T., & Gitas, I. Z. (2022). Assessing the Accuracy of MODIS MCD64A1 C6 and FireCCI51 Burned Area Products in Mediterranean Ecosystems. *Remote Sensing*, *14*(3), 1–15. https://doi.org/10.3390/rs14030602

Kukavskaya, E. A., Ivanova, G. A., Conard, S. G., McRae, D. J., & Ivanov, V. A. (2014). Biomass dynamics of central Siberian Scots pine forests following surface fires of varying severity. *International Journal of Wildland Fire*, *23*(6), 872–886. https://doi.org/10.1071/WF13043

Kukavskaya, E. A., Shvetsov, E. G., Buryak, L. V., Tretyakov, P. D., & Groisman, P. Y. (2023). Increasing Fuel Loads, Fire Hazard, and Carbon Emissions from Fires in Central Siberia. *Fire*, *6*(2). https://doi.org/10.3390/fire6020063

Lizundia-Loiola, J., Franquesa, M., Boettcher, M., Kirches, G., Pettinari, M. L., & Chuvieco, E. (2021). Implementation of the burned area component of the copernicus climate change service: from modis to olci data. *Remote Sensing*, *13*(21). https://doi.org/10.3390/rs13214295

Lizundia-Loiola, J., Otón, G., Ramo, R., & Chuvieco, E. (2020). A spatio-temporal active-fire clustering approach for global burned area mapping at 250 m from MODIS data. *Remote Sensing of Environment*, *236*, 111493. https://doi.org/10.1016/J.RSE.2019.111493

Meijer, J. R., Huijbregts, M. A. J., Schotten, K. C. G. J., & Schipper, A. M. (2018). Global patterns of current and future road infrastructure. *Environmental Research Letters*, *13*(6). https://doi.org/10.1088/1748-9326/aabd42

Monteith, J., & Unsworth, M. (2013). Principles of Environmental Physics: Plants, Animals, and the Atmosphere: Fourth Edition. In *Principles of Environmental Physics: Plants, Animals, and the Atmosphere: Fourth Edition*. https://doi.org/10.1016/C2010-0-66393-0

Muñoz Sabater, J. (2019). *ERA5-Land hourly data from 1950 to present.* Copernicus Climate Change Service (C3S) Climate Data Store (CDS). https://doi.org/10.24381/cds.e2161bac

Olson, D. M., Dinerstein, E., Wikramanayake, E. D., Burgess, N. D., Powell, G. V. N., Underwood, E. C., D’amico, J. A., Itoua, I., Strand, H. E., Morrison, J. C., Loucks, C. J., Allnutt, T. F., Ricketts, T. H., Kura, Y., Lamoreux, J. F., Wettengel, W. W., Hedao, P., & Kassem, K. R. (2001). Terrestrial Ecoregions of the World: A New Map of Life on Earth. *BioScience*, *51*(11), 933. https://doi.org/10.1641/0006-3568(2001)051[0933:teotwa]2.0.co;2

Pekel, J. F., Cottam, A., Gorelick, N., & Belward, A. S. (2016). High-resolution mapping of global surface water and its long-term changes. *Nature*, *540*(7633), 418–422. https://doi.org/10.1038/nature20584

Rogers, B. M., Soja, A. J., Goulden, M. L., & Randerson, J. T. (2015). Influence of tree species on continental differences in boreal fires and climate feedbacks. *Nature Geoscience*, *8*(3), 228–234. https://doi.org/10.1038/ngeo2352

Santoro, M., Cartus, O., Carvalhais, N., Rozendaal, D. M. A., Avitabile, V., Araza, A., De Bruin, S., Herold, M., Quegan, S., Rodríguez-Veiga, P., Balzter, H., Carreiras, J., Schepaschenko, D., Korets, M., Shimada, M., Itoh, T., Moreno Martínez, Á., Cavlovic, J., Gatti, R. C., … Willcock, S. (2021). The global forest above-ground biomass pool for 2010 estimated from high-resolution satellite observations. *Earth System Science Data*, *13*(8). https://doi.org/10.5194/essd-13-3927-2021

Schroeder, W., & Giglio, L. (2018). *VIIRS/NPP Active Fires 6-Min L2 Swath 375m - LAADS DAAC*. https://ladsweb.modaps.eosdis.nasa.gov/missions-and-measurements/products/VNP14IMG/#overview

Sedano, F., & Randerson, J. T. (2014). Multi-scale influence of vapor pressure deficit on fire ignition and spread in boreal forest ecosystems. *Biogeosciences*, *11*(14), 3739–3755. https://doi.org/10.5194/bg-11-3739-2014

Takaku, J., Tadono, T., Doutsu, M., Ohgushi, F., & Kai, H. (2020). UPDATES OF ‘AW3D30’ ALOS GLOBAL DIGITAL SURFACE MODEL WITH OTHER OPEN ACCESS DATASETS. *The International Archives of the Photogrammetry, Remote Sensing and Spatial Information Sciences*, *XLIII*-*B4*-*2*(B4), 183–189. https://doi.org/10.5194/isprs-archives-XLIII-B4-2020-183-2020
